# Paraspinal Fat Quantity and Three-Dimensional Spatial Distribution in Relation to Isokinetic Trunk Strength: An Exploratory Cross-Sectional Study

**DOI:** 10.3390/jcm15218334

**Published:** 2026-10-26

**Authors:** Fangyi Wei, Jiahua Feng, Zhongning Xu, Jintao Ao, Tenghui Ge, Jingye Wu, Zhao Lang, Ling Wang, Yuqing Sun

**Affiliations:** 1 Department of Spine Surgery, Beijing Jishuitan Hospital, Capital Medical University, Beijing 100035, China; f_wei939@163.com (F.W.); 2025fengjh@mail.ccmu.edu.cn (J.F.);; 2 National Center for Orthopaedics, Beijing 100035, China; 3 Department of Spine Surgery, Beijing Jishuitan Hospital, Fourth Clinical College of Peking University, Beijing 100035, China; 4 JST Sarcopenia Research Centre, Beijing Research Institute of Traumatology and Orthopaedics, Beijing Jishuitan Hospital, Beijing 100035, China; 5 Department of Radiology, Beijing Jishuitan Hospital, Capital Medical University, Beijing 100035, China

**Keywords:** paraspinal muscles, fat infiltration, centroid offset, computed tomography, isokinetic trunk strength, rehabilitation

## Abstract

**Background/Objectives:** Fat infiltration describes how much fat is present but not its location within a muscle compartment. This exploratory study characterized regional fat distribution in older adults and explored whether spatial descriptors provide information about trunk strength beyond fat infiltration (FI). **Methods:** 79 community-dwelling healthy adults underwent CT-based thoracic and lumbar posterior paraspinal assessment and isokinetic strength testing. Fat infiltration and direction-corrected, size-normalized fat-to-whole-compartment centroid offsets were examined using correlations and multivariable linear regression adjusted for age, sex, and corresponding FI. **Results:** Fat centroids were consistently medial and predominantly anterior relative to their compartment references, with superior thoracic and inferior lumbar positions. Right-sided FI was higher than left-sided FI, with mean differences of 1.93 percentage points in the lumbar region and 0.58 percentage points in the thoracic region. Higher FI was associated with smaller medial offsets. Larger medial offsets tended to accompany greater extension and flexion strength after adjustment, with global medial offset providing additional explanatory information beyond FI. Adding this feature increased the explained variation in extension and flexion strength by approximately 6.7 and 6.2 percentage points, respectively. The positive medial-offset pattern was retained across sensitivity analyses, although no primary offset association met the false discovery rate criterion. **Conclusions:** Posterior paraspinal fat showed distinct regional spatial patterns and bilateral differences in fat quantity. Medial centroid offsets emerged as candidate features for functional assessment alongside FI. However, the positive medial-offset estimates did not establish strength associations after multiplicity adjustment, and inference was sensitive to covariance specification. Potential applications to functional assessment and rehabilitation monitoring require prospective validation.

## 1. Introduction

Posterior paraspinal muscles contribute to trunk movement and spinal stabilization. Their composition and function vary with aging and spine-related disorders, including intramuscular fat accumulation and changes in muscle morphology [1,2]. The multifidus and erector spinae have distinct anatomical locations, architectures, and mechanical roles [3,4,5]. Spinal stability also depends on coordination between passive structures, active muscles, and neural control, rather than on muscle morphology alone [6].

Muscle changes in low back pain are heterogeneous and can differ between acute, recurrent, and chronic presentations [7]. Systematic reviews have identified differences in paraspinal muscle size between pain and control populations, while also highlighting inconsistency, muscle specificity, and methodological limitations [8,9]. Community-based work has reported associations of paraspinal fat with pain, disability, and structural abnormalities, without establishing a causal direction [10]. This supports assessing composition alongside function but cautions against treating a fat measurement as a direct marker of impairment.

CT attenuation and MRI-derived fat fractions are commonly used to characterize paraspinal composition, but imaging methods and measurement protocols are not interchangeable [11,12]. MRI studies have linked fat fraction or functional muscle morphology to isometric strength in healthy subjects [13,14,15]. An overall fat proportion, however, describes how much fat is present rather than where it is located. Vertebral-level and regional variation can be obscured by a single averaged value [2,16].

Spatial methods have already extended assessment beyond overall quantity. Khattab et al. mapped fat relative to the lumbar disc center of rotation and identified muscle- and clinical-context-specific patterns [17]. Three-dimensional spatial parametric mapping subsequently demonstrated muscle-specific distributions and spatially varying associations with age, body mass index, and sex [18]. Large-cohort research also identifies age, sex, body mass index, and physical activity as relevant correlates of paraspinal composition [19]. The functional relevance of compact centroid-based measures spanning thoracic and lumbar compartments remains insufficiently characterized, particularly in relation to relative isokinetic trunk torque.

Isokinetic dynamometry assesses dynamic torque at a controlled angular velocity and complements isometric testing. Test position, velocity, and participant characteristics influence measurement interpretation and reliability [20]. Pairing volumetric imaging with body-weight-normalized extension and flexion torque can help determine whether spatial features remain associated with strength after accounting for age, sex, and corresponding FI.

This exploratory study aimed to characterize thoracic and lumbar fat quantity and three-dimensional centroid offsets; examine their correlations with relative isokinetic trunk strength; and identify age-, sex-, and FI-adjusted spatial associations for hypothesis generation. The emphasis was on spatial patterns, effect direction and magnitude, and estimation uncertainty.

## 2. Materials and Methods

### 2.1. Study Design and Participants

**Figure 1:**
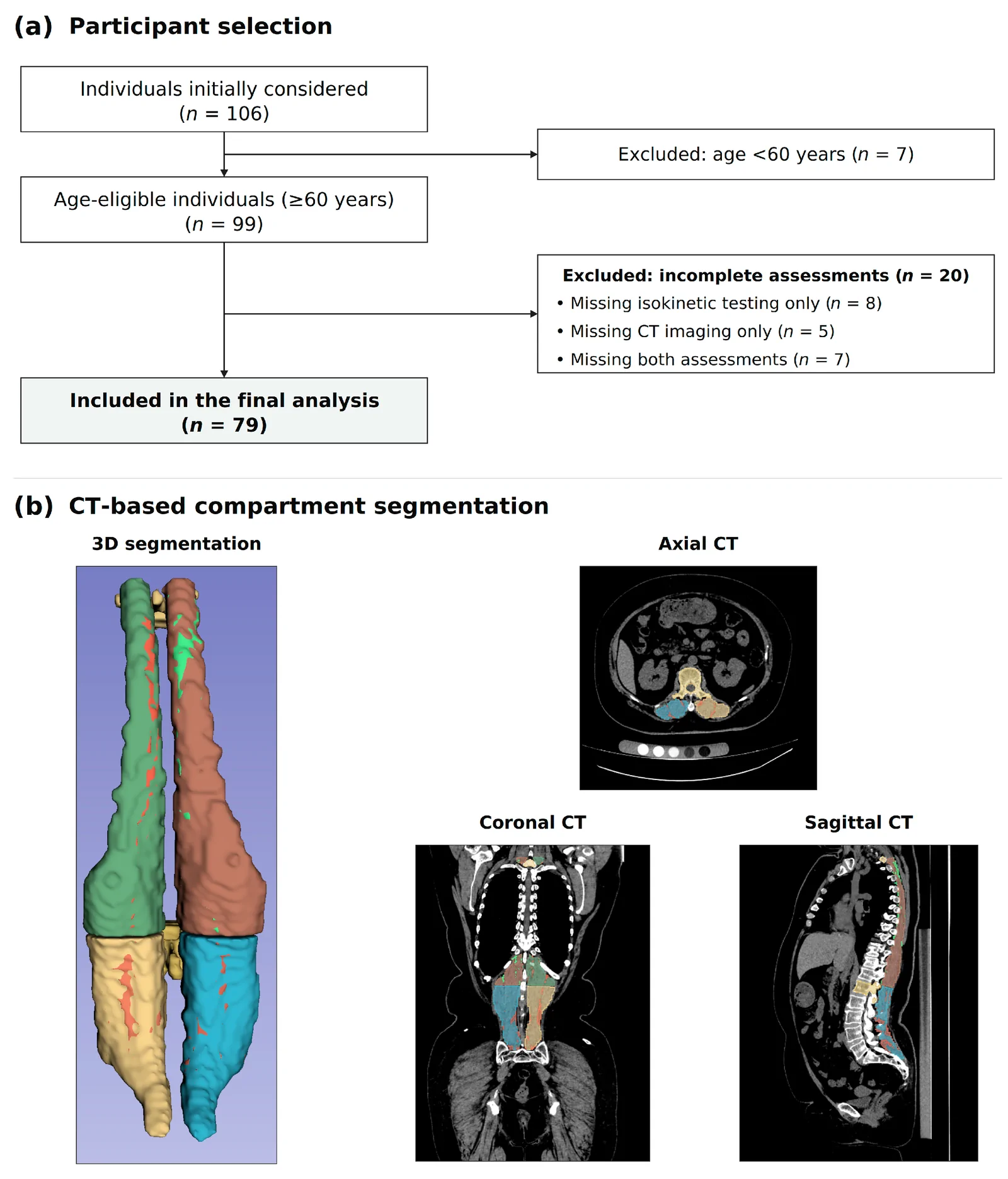
Study flowchart of participant inclusion and exclusion (**a**) and CT Segmentation (**b**). C7-T1 and T12-L1 disc levels served as dividing planes to create left/right thoracic and lumbar subregions shown in different colors. Fat is extracted within the fat-inclusive whole-compartment mask using a HU threshold of −190 to −30 HU. For instance, the axial image in (**b**) shows the left lumbar region in yellow, the right lumbar region in blue, and fat voxels within both compartments in red; the coronal and sagittal images show the left thoracic region in green and the right thoracic region in reddish-brown; fat voxels inside the left thoracic region are marked in red, while those within the right thoracic region are marked in light green; multifidus and erector spinae are not separately analyzed.

This was a retrospective cross-sectional analysis nested within an ongoing prospective cohort project at Beijing Jishuitan Hospital, Beijing, China. Participants were recruited from the community between August 2025 and January 2026. The eligibility criteria recorded in the original study protocol were age 60 years or older, either sex, written informed consent, independent ambulation and autonomous behavioral capacity, and willingness to attend follow-up visits. Exclusion criteria were previous spinal surgery; congenital spinal deformity; neuromuscular disease; rheumatic or immune disease, including ankylosing spondylitis; spinal tumors, infection, or fractures; substantial spinal cord compression; severe systemic disease; and cognitive or communication disorders. Because isokinetic testing was optional, the present analysis was restricted to participants with both CT imaging and isokinetic trunk-strength measurements available. In addition, CT and isokinetic testing were arranged to be performed on the same day. Of 106 individuals initially considered for the study, seven were excluded for being younger than 60 years, leaving 99 eligible individuals. Among these, 15 declined or were unable to undergo isokinetic testing, and CT imaging data were unavailable for 12. Seven participants did not complete both CT imaging and isokinetic testing. The final analysis dataset contained 79 participants (study flowchart in Figure 1). Manuscript organization was guided by STROBE reporting recommendations [21].

### 2.2. CT Acquisition and Compartment Segmentation

Participants underwent supine unenhanced chest and abdominal CT using a single scanner at Beijing Jishuitan Hospital. CT examinations were performed using an Aquilion PRIME scanner (Toshiba, Otawara, Japan) at a tube voltage of 120 kVp, with participants positioned supine, head first. The CT volume used for segmentation covered the thoracic and lumbar spine, with sagittal reconstructions extending inferiorly to the coccyx. Segmentation and quantitative analysis used the “Spine 1.0” DICOM series, reconstructed with the FC08 kernel at a slice thickness of 1 mm and a reconstruction interval of 0.8 mm. The in-plane pixel spacing was approximately 0.976 × 0.976 mm.

Images were imported into 3D Slicer, version 5.6.2. Automatic segmentation was performed with the TotalSegmentator extension (Version 76aaa53 (2025-01-07)) [22]. One experienced researcher visually inspected the masks and used the Segment Editor module for manual correction when needed. The corrections addressed inclusion of structures outside the posterior paraspinal compartment, omission of identifiable portions of the target compartment, and boundary deviations. The output segments label “Left deep back muscle” and “Right deep back muscle” were used as the initial masks for the left and right posterior paraspinal compartments, respectively. These masks were subsequently reviewed and manually corrected as described before, and then divided at a plane passing through the T12–L1 intervertebral disc, and the Keep selected island function was used to isolate the thoracic and lumbar components. Specifically, the dividing plane is defined as the mid-axial slice parallel to T12-L1 disc level (shown in Figure 1). Likewise, C7-T1 intervertebral disc level served as superior dividing plane. This yielded four subregions: thoracic left, thoracic right, lumbar left, and lumbar right (Figure 1).

Fifteen participants were randomly selected for repeatability assessment. Both raters reviewed and made localized manual corrections to the same automatically generated initial segmentation, rather than performing independent manual segmentations from scratch. The same rater repeated assessment under blinded conditions approximately one month later. FI and normalized centroid offsets were recalculated. Two-way random-effects, absolute-agreement, single-measure ICC (2,1) with 95% confidence intervals was adopted to evaluate intra-observer and inter-observer measurement repeatability. Metric-specific ICCs, SEM and MDC95 are reported in Table S12.

Fat voxels within the whole-compartment masks were identified using an attenuation threshold of −190 to −30 HU. CT attenuation is a composition-related measure rather than a direct measurement of muscle function [23]. Fat-inclusive total compartment volume, fat volume, and their three-dimensional centroid coordinates were exported. The reference masks and reference centroids included intramuscular fat; they were not lean-muscle-only masks.

### 2.3. Fat Infiltration

Side-specific FI was defined as fat volume divided by fat-inclusive total compartment volume, multiplied by 100%. Regional FI was calculated from the summed left and right fat volumes divided by their summed total volumes. Global FI was the corresponding ratio across all four subregions. FI was therefore volume-weighted through ratios of summed volumes, rather than obtained by averaging side-specific percentages. Volumes were expressed in cm^3^ and FI in percent.

(1) $${\mathrm{FI}}_{g,d}=\frac{V_{g,d}^{\mathrm{fat}}}{V_{g,d}^{\mathrm{tot}}}\times 100\%$$

(2) $${\mathrm{FI}}_{g}=\frac{V_{g,L}^{\mathrm{fat}}+V_{g,R}^{\mathrm{fat}}}{V_{g,L}^{\mathrm{tot}}+V_{g,R}^{\mathrm{tot}}}\times 100\%$$

(3) $${\mathrm{FI}}_{G}=\frac{\sum_{g\in \{\mathrm{Th},\mathrm{Lu}\}}\sum_{d\in \{L,R\}}V_{g,d}^{\mathrm{fat}}}{\sum_{g\in \{\mathrm{Th},\mathrm{Lu}\}}\sum_{d\in \{L,R\}}V_{g,d}^{\mathrm{tot}}}\times 100\%$$

In Equations (1)–(3), g ∈ {Th, Lu} denotes the thoracic or lumbar region, and d ∈ {L, R} denotes the left or right side. V^fat^ and V^tot^ are fat volume and fat-inclusive whole-compartment volume in cm^3^. G denotes the global level. FI is a percentage: regional and global FI use ratios of summed volumes, not arithmetic means of side-specific FI.

### 2.4. Direction-Corrected and Normalized Fat Centroid Offsets

For each subregion, the raw centroid offset, denoted by the vector Δ^RAS^, was calculated as the fat centroid minus the fat-inclusive whole-compartment centroid in the original RAS coordinate system (Equation (4)). Exported coordinates followed the right–anterior–superior (RAS) convention, with right, anterior, and superior positive. The lateral difference was multiplied by −1 on the right side and left unchanged on the left side to make medial displacement positive. Anterior–posterior and superior–inferior differences were multiplied by −1 on both sides to make posterior and inferior displacement positive. The direction-corrected components were denoted by Δ^dir^ (Equation (5)); both the raw and direction-corrected differences were expressed in mm.

Each direction-corrected component Δ^dir^ was divided by the subregion-specific characteristic length ℓ, defined as 10 times the cube root of total volume in cm^3^. The factor of 10 converts the characteristic length to mm; the resulting normalized offsets, denoted by Δ with a tilde, are dimensionless (Equations (6) and (7)). Regional offsets were equal-weight averages of the two normalized side-specific values, and global offsets were equal-weight averages across all four subregions. They were not weighted by fat volume. Nine measures were generated: Δr, Δa, and Δs at lumbar, thoracic, and global levels. The term offset denotes a positional difference between two centroids, not tissue movement over time or distance to the anatomical midline.

(4) $$\Delta_{g,d}^{\mathrm{RAS}}=c_{g,d}^{\mathrm{fat}}-c_{g,d}^{\mathrm{whole}}\;={\left(\Delta_{g,d,r}^{\mathrm{RAS}},\;\Delta_{g,d,a}^{\mathrm{RAS}},\;\Delta_{g,d,s}^{\mathrm{RAS}}\right)}^{T}$$

Here, c^fat^ and c^whole^ denote the three-dimensional centroids of fat and the fat-inclusive whole compartment, respectively, expressed in mm in the right–anterior–superior (RAS) coordinate system. Their difference, the vector Δ^RAS^, is the raw centroid offset before anatomical direction correction or normalization. The superscript RAS identifies the original coordinate convention. The r, a, and s components are positive toward the right, anterior, and superior directions, respectively. The superscript T denotes vector transpose; g and d identify the region and side as defined above.

(5) $$\begin{array}{r} \Delta_{g,L,r}^{\mathrm{dir}}=\Delta_{g,L,r}^{\mathrm{RAS}},\;\;\Delta_{g,R,r}^{\mathrm{dir}}=-\Delta_{g,R,r}^{\mathrm{RAS}}\\ \Delta_{g,d,a}^{\mathrm{dir}}=-\Delta_{g,d,a}^{\mathrm{RAS}},\;\;\Delta_{g,d,s}^{\mathrm{dir}}=-\Delta_{g,d,s}^{\mathrm{RAS}} \end{array}$$

Δ^dir^ denotes the direction-corrected centroid difference obtained from Δ^RAS^; the superscript dir denotes anatomical direction correction. The axis index is k ∈ {r, a, s}. L and R in the side index mean left and right, not lumbar region. The corrected r, a, and s components are positive toward medial, posterior, and inferior directions, respectively; negative values indicate the opposite. These corrected differences remain in mm.

(6) $$l_{g,d}=10{\left(V_{g,d}^{\mathrm{tot}}\right)}^{1/3}\;\left(\mathrm{mm}\right)$$

(7) $${\tilde{\Delta }}_{g,d,k}=\frac{\Delta_{g,d,k}^{\mathrm{dir}}}{l_{g,d}}$$

ℓ_g,d_ is the characteristic length in mm. Because V^tot^ is measured in cm^3^, its cube root is in cm, and multiplication by 10 converts it to mm. The tilde on Δ identifies a normalized, dimensionless offset, obtained by dividing the direction-corrected component Δ^dir^ by the characteristic length of that same subregion. Thus, Δ^RAS^ is the raw coordinate difference in mm, Δ^dir^ is the direction-corrected difference in mm, and Δ with a tilde is the normalized offset without units.

(8) $${\tilde{\Delta }}_{g,k}=\frac{{\tilde{\Delta }}_{g,L,k}+{\tilde{\Delta }}_{g,R,k}}{2}$$

(9) $${\tilde{\Delta }}_{G,k}=\frac{1}{4}\sum_{g\in \{\mathrm{Th},\mathrm{Lu}\}}\sum_{d\in \{L,R\}}{\tilde{\Delta }}_{g,d,k}$$

**Figure 2:**
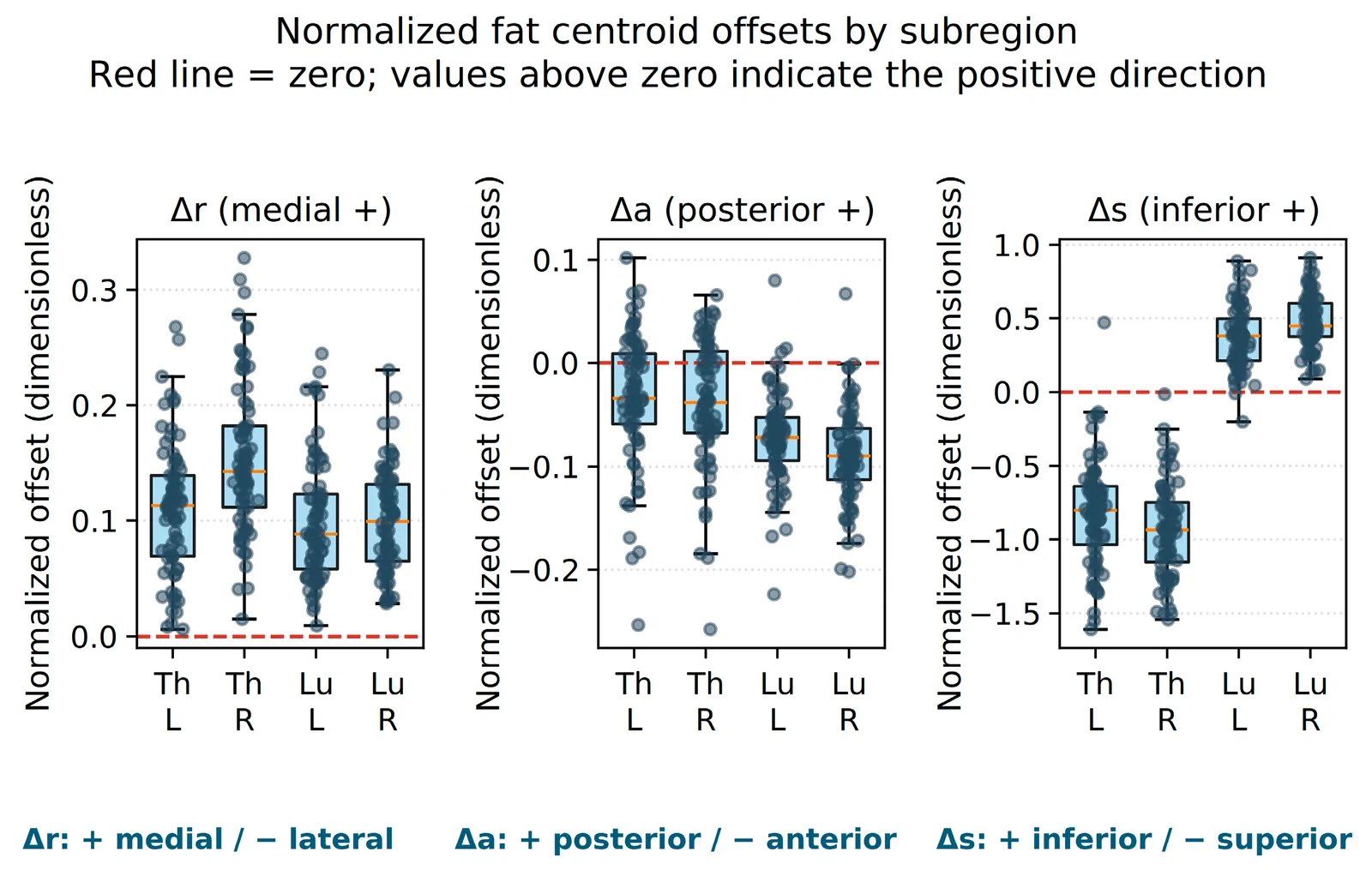
Normalized fat-centroid offsets in four side-specific subregions (*n* = 79). Th/Lu indicate thoracic/lumbar regions and L/R left/right sides. Positive Δr/Δa/Δs indicate medial/posterior/inferior positions relative to the fat-inclusive whole-compartment centroid; negative values indicate lateral/anterior/superior positions. Boxes show Q1–Q3, central lines the median, and whiskers observations within 1.5 interquartile ranges. Points are participants. The red line is zero. Offsets are dimensionless; Δr is medial-positive on both sides. These signs differ from uncorrected RAS directions.

The regional normalized offset is the equal-weight average of the left and right values. The global offset is the equal-weight average of the four normalized subregions. It is not recalculated by dividing a pooled raw difference by a global volume-derived length. In the Results, tables, and figures, Δr, Δa, and Δs are shorthand for these normalized offsets; the tilde is omitted for readability. Δ^RAS^ and Δ^dir^ are reserved for the unnormalized raw and direction-corrected differences, respectively. For quick interpretation, positive Δr, Δa, and Δs indicate medial, posterior, and inferior fat-centroid positions relative to the whole-compartment centroid; negative values indicate lateral, anterior, and superior positions, respectively. The direction key in Figure 2 and the schematic in Figure S5 summarize these signs. The axis labels r, a, and s are retained from RAS notation but do not denote the original positive RAS directions after correction.

For a reference-centroid sensitivity analysis, the centroid of the non-fat complement was reconstructed (detailed calculations in Table S9). Corrected offsets were computed against this reference and normalized before equal-weight averaging. The complement is not a separately segmented lean-muscle compartment, and the analysis does not provide independent measurement validation.

### 2.5. Isokinetic Trunk Strength Testing

Trunk flexion and extension peak torques were measured using a BioniX Sim3 Pro dynamometer (Physiomed, Schnaittach, Germany). The testing apparatus and setup are illustrated in Figure S1. Participants with a visual analogue scale (VAS) < 3 for leg or back pain were tested in the standing position. The pelvis was firmly stabilized by adjustable padded clamps positioned over the anterior superior iliac spines, and additional supports were applied to the upper thorax and shoulders according to the manufacturer’s positioning workflow. The dynamometer flexion-extension rotational axis was aligned to the anatomical center of the L3-L4 spinal segment guided by the built-in laser alignment marker. Individual active trunk range-of-motion (ROM) was determined for each participant prior to formal testing: participants performed slow, pain-limited flexion-extension movements with the axis unlocked, and the personalized ROM was recorded and accepted within the device software, constrained between upright neutral (0°) and 45° trunk flexion. Following a standardized familiarization trial, participants completed five maximal voluntary flexion-extension repetitions; 60 s rest was provided between repetitions. Gravity correction was applied by the system software to eliminate torque contributions originating from upper-trunk mass and fixture attachments. Peak flexion and extension torque values were extracted for each valid repetition, and the maximum peak-torque value across trials was retained for statistical analysis. The 15°/s isokinetic velocity was selected to facilitate slow, well-controlled trunk movement in older adults and reduce risk of uncontrolled rapid motion. Published reliability evidence for BioniX-based trunk isokinetic testing predominantly originates from younger populations examined at higher angular velocities (60°/s, 90°/s); therefore, those reports cannot directly validate our 15°/s testing condition [24]. Therefore, reliability data for this exact 15°/s standing trunk protocol in older adults are not available in the present study.

The outcomes were body-weight-normalized extension and flexion peak torques (Nm/kg), obtained directly from the dynamometer output. The normalization relationship is:

(10) $$T_{\mathrm{rel}}=\frac{T_{\mathrm{abs}}}{\mathrm{BW}}\;\left(\mathrm{Nm}/\mathrm{kg}\right)$$

T_rel_ is relative peak torque in Nm/kg, T_abs_ is absolute peak torque in Nm, and BW is body weight in kg. The relationship is applied separately to extension and flexion. The dynamometer reports relative peak torque rounded to one decimal place. These device-exported rounded values were used directly in the primary analyses. Ratios recalculated from the recorded absolute peak torque and body weight, without additional rounding, were used in sensitivity analysis to assess the influence of rounding precision (Table S7C).

### 2.6. Statistical Analysis

Participant-characteristic distributions were assessed using Shapiro–Wilk tests together with histograms and normal Q–Q plots. Approximately normal variables without marked skewness were summarized as mean ± SD; other continuous variables were summarized as median (Q1–Q3), and categorical variables as *n* (%). Bilateral comparisons used paired t tests and Wilcoxon signed-rank tests (Table S1, panel B). Unadjusted associations with each relative torque outcome were assessed using Spearman correlations across 16 demographic, FI, and offset variables. These tests received BH correction within each 16-test outcome family.

Multivariable linear regression was used to examine associations between normalized offsets and each relative torque outcome. Each of nine separate models included age, sex (male = 1; female = 0), corresponding regional FI, and one normalized offset. M1–M3 represented lumbar Δr, Δa, and Δs; M4–M6 represented thoracic offsets; and M7–M9 represented global offsets. All nine offsets were not entered simultaneously. Body weight was not additionally included because outcomes were already weight-normalized. Nonstandardized B and standardized β were reported with 95% confidence intervals on their respective scales; β equals B multiplied by predictor SD and divided by outcome SD. Standard errors and t-based intervals were obtained from the fitted linear models. Complete-case models included 79 participants. Adjusted R^2^ was reported as a descriptive measure of fit for the entire model, including all covariates and the modeled offset. Residual patterns, heteroscedasticity, collinearity, and influential observations were examined as model checks.

BH-FDR correction was applied separately to the nine offset coefficients for each strength outcome, with q < 0.05 as the significance criterion. Model assumptions and influential observations were assessed using standard residual diagnostics, VIFs, leverage, and Cook’s distance. Sensitivity analyses examined HC3 standard errors, additional body-size and compartment-volume adjustment, alternative torque and offset definitions, volume weighting, and non-fat references. Model-specific influence checks excluded observations with Cook’s distance > 4/*n*; all eligible participants were retained in the primary analyses. Analyses were performed using Python 3.11.

(11) $$\begin{array}{r} Y_{i}=b_{0}+b_{\mathrm{age}}\;{\mathrm{Age}}_{i}+b_{\mathrm{sex}}\;{\mathrm{Sex}}_{i}\\ \;\;+b_{\mathrm{FI}}\;{\mathrm{FI}}_{h,i}+b_{\Delta }\;{\tilde{\Delta }}_{h,i,k}+\varepsilon_{i} \end{array}$$

i indexes participants, and Y_i_ is extension or flexion relative peak torque. h identifies the modeled level (Lu, Th, or G), and k identifies the one offset axis entered in that model. Age is in years, sex is coded male = 1 and female = 0, and FI_h,i_ is the corresponding FI in percent. b_0_ is the intercept; b_age_, b_sex_, b_FI_, and b_Δ_ are nonstandardized slopes (reported as B in the coefficient tables); ε_i_ is the error term. The model is fitted separately for each outcome, level, and axis.

(12) $$\beta_{j}=b_{j}\;\frac{s_{X_{j}}}{s_{Y}}$$

For continuous predictor X_j_, b_j_ is its nonstandardized slope, S_Xj_ and S_Y_ are the sample SDs of that predictor and outcome, and β_j_ is the standardized coefficient. Standardized 95% CIs correspond to the same coefficient scale; they must not be replaced by B-scale CIs.

### 2.7. Side-Specific Exploratory Analyses

Side-specific multivariable linear regression models entered left and right normalized offsets simultaneously with age, sex, and corresponding regional FI. The two sides were measurements from the same participants, not independent groups.

(13) $$\begin{array}{r} Y_{i}=b_{0}+b_{\mathrm{age}}\;{\mathrm{Age}}_{i}+b_{\mathrm{sex}}\;{\mathrm{Sex}}_{i}+b_{\mathrm{FI}}\;{\mathrm{FI}}_{g,i}\\ \;\;+b_{L}\;{\tilde{\Delta }}_{g,L,i,k}+b_{R}\;{\tilde{\Delta }}_{g,R,i,k}+\varepsilon_{i} \end{array}$$

This supplementary model uses a thoracic or lumbar region g. The left and right normalized offsets are entered simultaneously for the same participant i. b_L_ and b_R_ are their conditional nonstandardized slopes after accounting for the other side, age, sex, and regional FI. The remaining symbols have the meanings given for the main model. The two sides are not separate participant groups.

## 3. Results

### 3.1. Participant Characteristics

The analysis included 79 participants aged 60–79 years: 42 men (53.2%) and 37 women. Median age was 69.00 (68.00–73.00) years. Mean height and body weight were 163.32 ± 7.79 cm and 66.42 ± 11.25 kg. Median relative peak torques were 0.90 (0.70–1.30) Nm/kg for extension and 0.70 (0.50–0.90) Nm/kg for flexion. Median thoracic, lumbar, and global FI were 8.47%, 9.06%, and 8.71%, respectively (Table 1). Right-sided FI exceeded left-sided FI in both regions (Table S1, panel B); Of note, this is an observed compartment-level asymmetry, not evidence of a muscle-specific process.

### 3.2. Direction-Specific Spatial Distribution and Relationships with FI

After size normalization, Δr was positive in every subregion of every participant, indicating a consistently medial fat centroid relative to the whole-compartment centroid. Δa was predominantly negative, indicating an anterior position. Δs was predominantly negative (superior) in the thoracic region and positive (inferior) in the lumbar region (Figure 2; Table S1). Bilateral Δr correlations were r = −0.209 for thoracic and r = 0.524 for lumbar regions. Δa/Δs bilateral correlations ranged from 0.707 to 0.837 (Figure S2). These correlations describe between-side covariation, not intra- or inter-rater reliability.

In supplementary Pearson analyses, corresponding FI was negatively related to Δr in lumbar, thoracic, and global regions (r = −0.493, −0.425, and −0.568, respectively; all *p* < 0.001; Figure S3). A sensitivity analysis using the algebraically reconstructed non-fat complement centroid as the reference was performed to assess the dependence of the FI-Δr relationship on reference-centroid definition (Table S9).

### 3.3. Unadjusted Associations with Relative Trunk Strength

The unadjusted Spearman results are presented with effect sizes, *p* values, and secondary outcome-specific q values in Table 2. FI point estimates were negative for both torque outcomes. Several flexion correlations, including FI and lumbar/global Δr, met the secondary 16-test FDR criterion, whereas the extension correlation family did not. The explanatory-variable matrix is shown in Figure S4.

### 3.4. Exploratory Adjusted Offset and FI Associations

**Figure 3:**
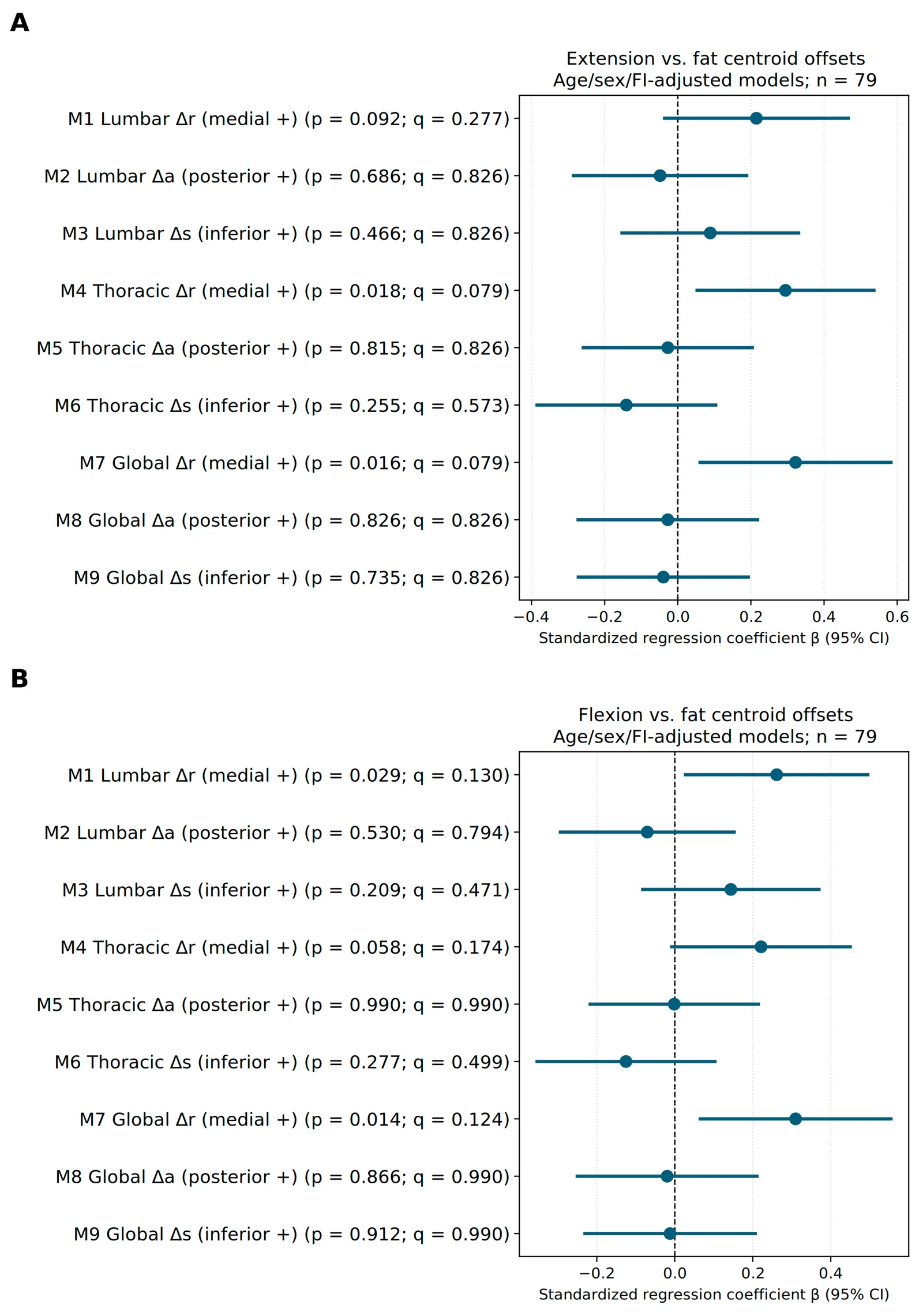
Adjusted associations between normalized offsets and relative trunk peak torque. (**A**) Extension; (**B**) flexion. *n* = 79. Each row is one separate age + sex + corresponding FI + offset model. Points and lines show standardized β and conventional t-based 95% CIs; the vertical dashed line is zero. *p* and q are displayed, with BH correction across all nine offset coefficients for each outcome. Uniform color avoids significance-based selection. HC3 sensitivity intervals are reported in Table S6.

**Figure 4:**
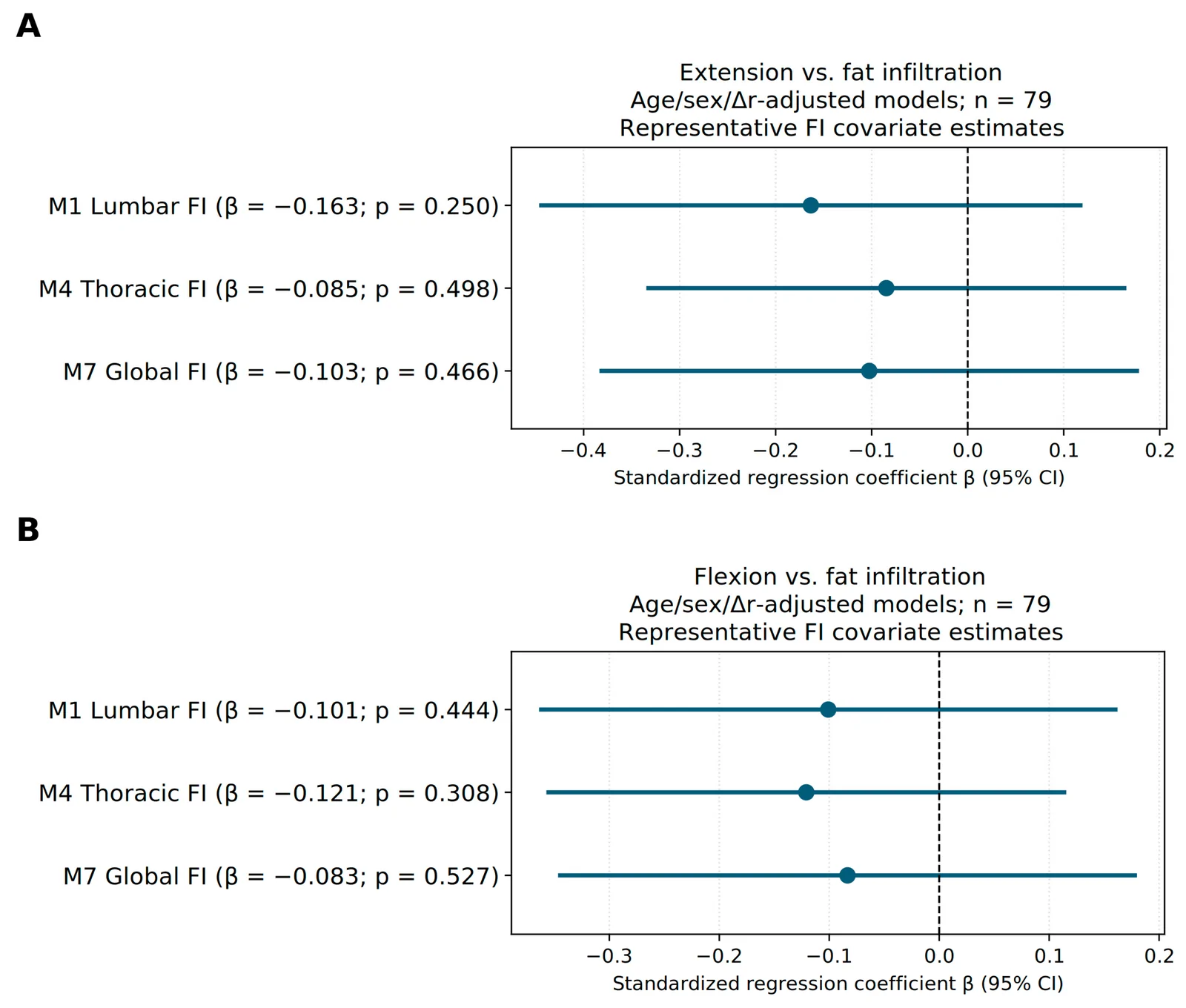
Representative FI covariate coefficients from Δr models, *n* = 79. (**A**), extension; (**B**), flexion. M1/M4/M7 correspond to lumbar/thoracic/global models adjusted for age, sex, and the indicated normalized Δr. Points and lines show standardized β and conventional 95% CIs. These model-context FI coefficients were not added to the nine-offset FDR families or selected by *p* value.

Adjusted Δr associations reached nominal significance for thoracic and global offsets during extension (β = 0.295 and 0.322; *p* = 0.018 and 0.016, respectively) and for lumbar and global offsets during flexion (β = 0.261 and 0.310; *p* = 0.029 and 0.014, respectively). Lumbar Δr during extension and thoracic Δr during flexion had *p* values between 0.05 and 0.10 (*p* = 0.092 and 0.058, respectively). However, none of the nine offset coefficients per outcome remained significant after BH-FDR correction, although Δr point estimates were consistently positive across regions and tasks. For global Δr, q values were 0.079 and 0.124 for extension and flexion, respectively; corresponding HC3 *p* values were 0.057 and 0.096, indicating sensitivity of inference to covariance specification. Full estimates and 95% confidence intervals are provided in Figure 3 and Table S2; representative FI covariate estimates are shown in Figure 4 and Table S3.

### 3.5. Side-Specific Patterns

Simultaneous side-specific models and direct slope contrasts are reported in Tables S4 and S11. The lumbar Δr left-minus-right contrast was *p* = 0.008 (q = 0.049) for extension and *p* = 0.036 (q = 0.216) for flexion. These secondary, model-dependent comparisons were not inferred from separate significance labels and do not establish reproducible biological lateralization.

### 3.6. Sensitivity Analyses

No offset association reached BH-FDR significance in the full-sample HC3, body-size, torque-definition, normalization, volume-weighting, or reconstructed-reference sensitivity analyses. In the model-specific influence-omission analysis, lumbar Δr was associated with flexion torque in a reduced subset (*n* = 71, q = 0.016). Full sensitivity results, diagnostic and influence checks, and incremental model-fit estimates are provided in Supplementary Tables S5–S10, with alternative offset definitions, normalization, and weighting analyses detailed in Table S8.

## 4. Discussion

### 4.1. Direction- and Region-Specific Fat-Centroid Positions

The principal contribution of this study is the description of direction- and region-specific fat-centroid positions within the combined posterior paraspinal compartments. Fat centroids were located medially relative to their whole-compartment references across the four subregions and were predominantly anterior. The superior–inferior pattern differed between regions: thoracic fat centroids were predominantly superior to their references, whereas lumbar fat centroids were inferior. These findings indicate that the spatial arrangement of compartmental fat cannot be adequately summarized by a single displacement direction.

Regional and level-dependent composition has been documented using MRI [2,16], while disc-centered mapping and three-dimensional spatial analyses demonstrate that fat occupies non-uniform locations [17,18]. The present three-axis descriptors extend this perspective across thoracic and lumbar compartments using a common normalization scheme. FI describes the proportion of a compartment classified as fat, whereas centroid offsets describe the relative position of that fat within the compartment. These measurements therefore address different aspects of morphology, although they are not mathematically independent.

The contrasting superior–inferior patterns demonstrate why regional descriptions remain important. Averaging thoracic and lumbar offsets can partially cancel opposing signs, producing a small global value despite distinct regional patterns. Global offsets should consequently be interpreted as summary measures rather than substitutes for regional measurements. Equal- and volume-weighted summaries emphasize different aspects of subregional data, and neither provides a complete representation of the spatial distribution.

These descriptors remain summaries of static anatomy. A centroid represents the first spatial moment of a volume and cannot distinguish a focal deposit from diffuse fat with the same centroid. The observed positions should therefore not be interpreted as movements of fat over time or as complete maps of three-dimensional heterogeneity.

### 4.2. Axis- and Region-Specific Relationships Between Fat Position and FI

Higher FI was associated with smaller medial centroid offsets in the lumbar, thoracic, and global measurements (r = −0.493, −0.425, and −0.568, respectively). Participants with greater fat burden therefore tended to show less medial separation between the fat centroid and the whole-compartment centroid. This finding links the amount of fat to its spatial position and supports describing paraspinal composition using both features. FI quantifies the proportion of fat, whereas centroid offsets characterize its anatomical position within the compartment. Together, they provide a more detailed morphological description than fat proportion alone.

The inverse FI–Δr relationship remained present when the reconstructed non-fat complement centroid was used as the reference, although the correlations were attenuated (Table S9). The overall descriptive pattern was therefore retained across reference definitions, while its magnitude depended partly on how the reference was constructed. This is relevant because fat contributes to the whole-compartment centroid: differences in FI can influence both the fat burden and the reference position.

Regional differences further support this combined assessment. The FI–anterior–posterior relationship was more apparent in the lumbar than in the thoracic compartment, while the contrasting superior thoracic and inferior lumbar fat positions highlighted distinct regional arrangements. A single global value may obscure these differences through averaging or cancellation. Retaining regional descriptors alongside FI could therefore help distinguish where otherwise similar overall fat burdens are located. For future clinical research, this offers a framework for examining whether regional fat patterns are associated with different functional profiles or longitudinal changes. The present findings describe differences between participants; their evolution over time remains a question for longitudinal studies.

### 4.3. Functional Relevance of Medial Fat Distribution and Bilateral Asymmetry

The functional analyses identified medial fat position as the most promising spatial feature for further investigation. Across thoracic, lumbar and global measurements, larger medial offsets tended to accompany greater extension and flexion peak torques after adjustment for age, sex, and corresponding FI. The clearest nominal associations involved thoracic and global Δr during extension, and lumbar and global Δr during flexion. However, none of the primary offset coefficients met the BH-FDR criterion. Adding global Δr increased the explained variation in extension and flexion strength by approximately 6.7 and 6.2 percentage points, respectively. These were descriptive, in-sample fit increments rather than validated improvements in predictive performance. These findings suggest that the spatial position of fat may contribute information about functional heterogeneity beyond the overall amount of fat, supporting further evaluation of FI and medial offsets as complementary imaging parameters.

Clinically, this distinction is relevant because a similar degree of FI can coexist with different spatial arrangements within the paraspinal compartment. As described above, FI summarizes fat burden, whereas centroid offsets show its relative positions. Their combination may provide a framework for investigating whether different morphological profiles correspond to different levels of trunk muscle strength. Because trunk torque integrates coordinated muscle activity, stabilization, and body geometry [3,6,20], muscle-specific morphology and localized functional measurements are needed to clarify their anatomical and functional basis.

The bilateral findings add another important dimension. Right-sided FI was higher than left in both regions, with a larger average difference in the lumbar region than in the thoracic region (1.93 versus 0.58 percentage points). Both paired differences remained significant after FDR correction. This consistent right-greater FI asymmetry may reflect inherent anatomical variation, habitual postural bias, or segmentation-related factors including partial-volume effects, but our cross-sectional data cannot fully separate these potential contributors. In contrast, larger lumbar Δr on the left was associated with higher torque (positive conditional coefficient), whereas the point estimate for right lumbar was negative. And the direct left-right slope contrast met the secondary FDR criterion for extension, but not for flexion (Table S11). Thus, the side with greater fat accumulation was not the side showing the clearest positive spatial-strength association. This distinction supports retaining bilateral FI and spatial measurements rather than reducing paraspinal morphology to a single side-averaged value.

Together, these observations prioritize global and regional medial offsets, particularly side-specific lumbar measurements, for prospective functional research. Incorporating these descriptors into existing CT assessments could enrich morphological phenotyping, while longitudinal studies could examine whether changes in fat position and bilateral asymmetry accompany changes in strength or rehabilitation outcomes. The primary offset associations did not meet the BH-FDR criterion, and the direct side contrasts were sensitive to analytical choices. These clinical applications therefore warrant further validation.

### 4.4. Anatomical Interpretation and Potential Relevance

Multifidus and erector spinae differ in architecture and mechanical roles [1,3,4,5], but the present workflow assessed their combined posterior compartment. A medial fat centroid may reflect the relative arrangement of these muscles, compartment shape and boundaries, and fat distribution. The current measurements cannot determine which individual muscle contains a fat voxel or reliably distinguish intramuscular from intermuscular adipose tissue. Moreover, centroid offset is not equivalent to muscle moment arm relative to the spinal joint axis [3,25].

Targeted exercise studies provide a relevant clinical context. Hides et al. reported fewer low back pain recurrences following specific multifidus and transversus abdominis retraining in patients with a first acute episode [26]. A Cochrane review subsequently found that the motor exercise improved some outcomes compared with minimal intervention, while providing outcomes similar to other exercise approaches [27]. More recently, a 12-week randomized trial in 50 patients with chronic non-specific low back pain is informative in this regard: combining motor control with isolated lumbar extension resistance training produced greater increases in multifidus and erector spinae cross-sectional area at L4–L5 and L5–S1 than general exercise, yet fat fraction did not improve in either arm and pain, disability, and quality of life improved comparably between groups, with no association between morphological and clinical change [28,29]. Of note, this trial assessed only level-specific, slice-based fat infiltration rather than volumetric measurements, which is an important limitation given the non-uniform spatial distribution of paraspinal fat described above. Thus, rehabilitation benefit need not necessarily be defined by reductions in intramuscular fat. It also supports assessment of muscle size, composition, and function as complementary outcomes.

Bayraktar et al. add a mechanical perspective relevant to older adults [30]. In women aged 60 years and older, multifidus and erector spinae stiffness measured using shear wave elastography was lower in the osteopenic group with vertebral fractures than in the group without fractures after adjustment for bone mineral density. Their findings highlight an association between paraspinal mechanical properties and spinal health. Combining muscle-specific stiffness measurements with CT-derived fat quantity and spatial organization could help determine whether particular morphological profiles accompany altered mechanical properties and functional performance.

### 4.5. Study Limitations and Validation Priorities

These findings are primarily hypothesis-generating. Effect direction and magnitude should be interpreted alongside confidence intervals, rather than treating *p* < 0.05 as definitive evidence. The cross-sectional design cannot establish temporal sequence, cause, prognosis, or treatment response.

The modest sample and confidence intervals limit precision. Single-center recruitment constrains generalizability, and other residual confounding might also need to be considered [19]. Centroids do not capture focal clustering or texture. Repeatability estimates apply to manual correction of a shared automatic initialization and do not validate independent-from-scratch segmentation. Test-retest reliability of the exact 15°/s standing protocol in older adults was not assessed. Physical activity and quantified degenerative spinal changes were not included in the reported adjustment set. These factors, together with residual body-size effects, may influence the observed associations.

Priorities are independent muscle-specific segmentation, localized functional assessment, and replication of the three-axis, FI, and Δr–trunk strength relationships in new samples. These steps would help distinguish a descriptive spatial phenotype from a clinically useful assessment or treatment-selection marker.

## 5. Conclusions

Posterior paraspinal fat in older adults exhibited consistent medial positioning, contrasting superior thoracic and inferior lumbar patterns, and higher right-sided FI. These findings support preserving regional and bilateral information when assessing paraspinal morphology. Although medial offsets had positive adjusted estimates for trunk strength, none of the primary offset coefficients met the BH-FDR criterion, and inference was sensitive to covariance specification. Spatial descriptors therefore warrant further investigation alongside FI, but their potential applications to functional assessment and rehabilitation monitoring remain proposals requiring prospective validation.

## Figures and Tables

**Table 1:** Participant characteristics, trunk strength, and paraspinal muscle morphology.

Characteristic	Overall (*n* = 79)
Age (years)	69.00 (68.00–73.00)
Male sex, *n* (%)	42 (53.2)
Height (cm)	163.32 ± 7.79
Body weight (kg)	66.42 ± 11.25
Relative extension peak torque (Nm/kg)	0.90 (0.70–1.30)
Relative flexion peak torque (Nm/kg)	0.70 (0.50–0.90)
Absolute extension peak torque (Nm)	61.10 (43.05–83.55)
Absolute flexion peak torque (Nm)	45.10 (32.75–59.75)
Thoracic FI (%)	8.47 (6.06–11.08)
Lumbar FI (%)	9.06 (6.92–13.26)
Global FI (%)	8.71 (6.85–13.19)
Thoracic bilateral total volume (cm^3^)	362.05 (300.50–424.99)
Lumbar bilateral total volume (cm^3^)	499.69 (441.17–560.83)

Height and body weight are mean ± SD; age, torque, FI, and compartment volumes are median (Q1–Q3); sex is *n* (%). Both continuous summaries and ranges are provided in Table S1. FI is calculated from summed fat volume divided by summed whole-compartment volume. Absolute torque values are the recorded torque measurements, not back-calculated from the rounded relative export. Total compartment volume includes fat. Nm, Newton-meters; FI, fat infiltration.

**Table 2:** Spearman correlations with relative trunk peak torque.

Variable	Ext. ρ	*p*	q	Flex. ρ	*p*	q
Age (years)	−0.232	0.040	0.107	−0.417	<0.001	0.002
Sex (male = 1)	0.072	0.528	0.592	0.129	0.259	0.319
Height (cm)	0.062	0.589	0.592	0.131	0.249	0.319
Body weight (kg)	−0.216	0.055	0.127	−0.098	0.390	0.446
Lumbar FI (%)	−0.283	0.011	0.081	−0.355	0.001	0.005
Thoracic FI (%)	−0.268	0.017	0.081	−0.362	0.001	0.005
Global FI (%)	−0.287	0.010	0.081	−0.376	<0.001	0.005
Lumbar Δr	0.241	0.033	0.104	0.305	0.006	0.017
Thoracic Δr	0.206	0.068	0.136	0.239	0.034	0.077
Global Δr	0.261	0.020	0.081	0.335	0.003	0.008
Lumbar Δa	−0.061	0.592	0.592	−0.058	0.609	0.609
Thoracic Δa	−0.165	0.145	0.211	−0.142	0.213	0.310
Global Δa	−0.173	0.127	0.203	−0.163	0.151	0.268
Lumbar Δs	0.117	0.305	0.407	0.144	0.205	0.310
Thoracic Δs	−0.179	0.114	0.202	−0.217	0.055	0.109
Global Δs	−0.068	0.552	0.592	−0.087	0.447	0.476

ρ is the Spearman rank coefficient (*n* = 79). Ext., extension; Flex., flexion. q values are BH-adjusted within all 16 correlations for each outcome, separately from the nine-offset regression families. *p* values are two-sided. Positive Δr/Δa/Δs indicate medial/posterior/inferior fat-centroid positions.

## Data Availability

The data presented in this study are available from the corresponding author upon reasonable request. The data are not publicly available due to patient privacy and institutional ethical restrictions.

## Supplementary Materials

The following supporting information can be downloaded at: https://www.mdpi.com/article/10.3390/jcm15218334/s1, Figures S1–S5 and Tables S1–S12 are supplied in the accompanying Supplementary Materials document.

## Abbreviations

The following abbreviations and statistical symbols are used in this manuscript:

**Abbreviation/symbol**	**Meaning**
CT	Computed tomography
MRI	Magnetic resonance imaging
FI	Fat infiltration
RAS	Original right–anterior–superior coordinate convention: positive directions are right, anterior, and superior, respectively; distinct from the anatomically corrected offset signs
Δ^RAS^	Raw fat-centroid minus fat-inclusive whole-compartment centroid vector in RAS coordinates (mm), before direction correction or normalization
Δ^dir^	Anatomically direction-corrected centroid difference (mm), before size normalization: positive components are medial, posterior, and inferior
Δr	Dimensionless, size-normalized medial–lateral fat-centroid offset relative to the whole-compartment centroid: positive = medial and negative = lateral on both sides
Δa	Dimensionless, size-normalized anterior–posterior fat-centroid offset relative to the whole-compartment centroid: positive = posterior and negative = anterior
Δs	Dimensionless, size-normalized superior–inferior fat-centroid offset relative to the whole-compartment centroid: positive = inferior and negative = superior
CI	Confidence interval
SD	Standard deviation
HU	Hounsfield units
VIF	Variance inflation factor
BW	Body weight
STROBE	Strengthening the Reporting of Observational Studies in Epidemiology
r	Pearson correlation coefficient
ρ	Spearman rank correlation coefficient
β	Standardized regression coefficient
