# Supplementary material for: Paraspinal Fat Quantity and Three-Dimensional Spatial Distribution in Relation to Isokinetic Trunk Strength: An Exploratory Cross-Sectional Study
Source: J Clin Med. 2026 Oct 26;15(21):8334. doi: 10.3390/jcm15218334 (PMC13764293; doi:10.3390/jcm15218334)
Supplement: Supplementary file 1 [file jcm-15-08334-s001.zip › jcm-4635068-supplementary.pdf]

Paraspinal Fat Quantity and Three-Dimensional Spatial Distribution in Relation to Isokinetic Trunk Strength: An Exploratory Cross-Sectional Study

Fangyi Wei [1,2], Jiahua Feng [1,2], Zhongning Xu [2,3], Jintao Ao [2,3], Tenghui Ge [2,3], Jingye Wu [2,3], Zhao Lang [1,2], Ling Wang [4,5,*] and Yuqing Sun [1,2,3,*]

[1] Department of Spine Surgery, Beijing Jishuitan Hospital, Capital Medical University, Beijing 100035, China; f_wei939@163.com (F.W.); 2025fengjh@mail.ccmu.edu.cn (J.F.); lorchid_jst@163.com (Z.L.)

[2] National Center for Orthopaedics, Beijing 100035, China; xuzhongning111@pku.edu.cn (Z.X.); 2311110591@pku.edu.cn (J.A.); 18811035656@163.com (T.G.); wujingyewiseman@sina.com (J.W.)

[3] Department of Spine Surgery, Beijing Jishuitan Hospital, Fourth Clinical College of Peking University, Beijing 100035, China

[4] JST Sarcopenia Research Centre, Beijing Research Institute of Traumatology and Orthopaedics, Beijing Jishuitan Hospital, Beijing 100035, China

[5] Department of Radiology, Beijing Jishuitan Hospital, Capital Medical University, Beijing 100035, China

[*] Correspondence: doctorwl@bjmu.edu.cn (L.W.); sunyuqing@jst-hosp.com.cn (Y.S.); Tel.: +86-18810527004 (L.W.); +86-13691303767 (Y.S.)

These Supplementary Materials contain Figures S1–S5 and Tables S1–S12 for the 79 age-eligible participants. Measurement repeatability was assessed in 15 participants using a shared automatic-initialization/manual-correction workflow (Table S12). Primary offset BH families contain nine tests per torque outcome. Additional analyses are exploratory checks in the same study cohort, not independent validation.

Figure S1

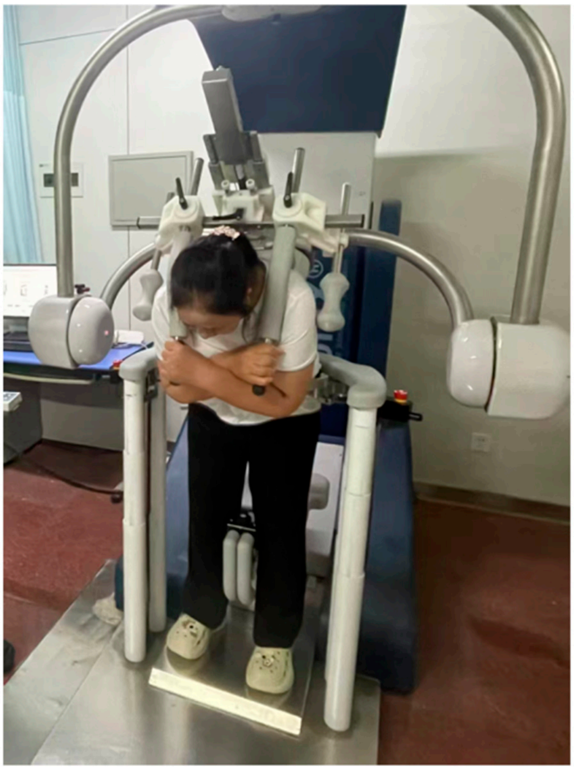

Figure S1. Isokinetic testing apparatus and test illustration.

Figure S2

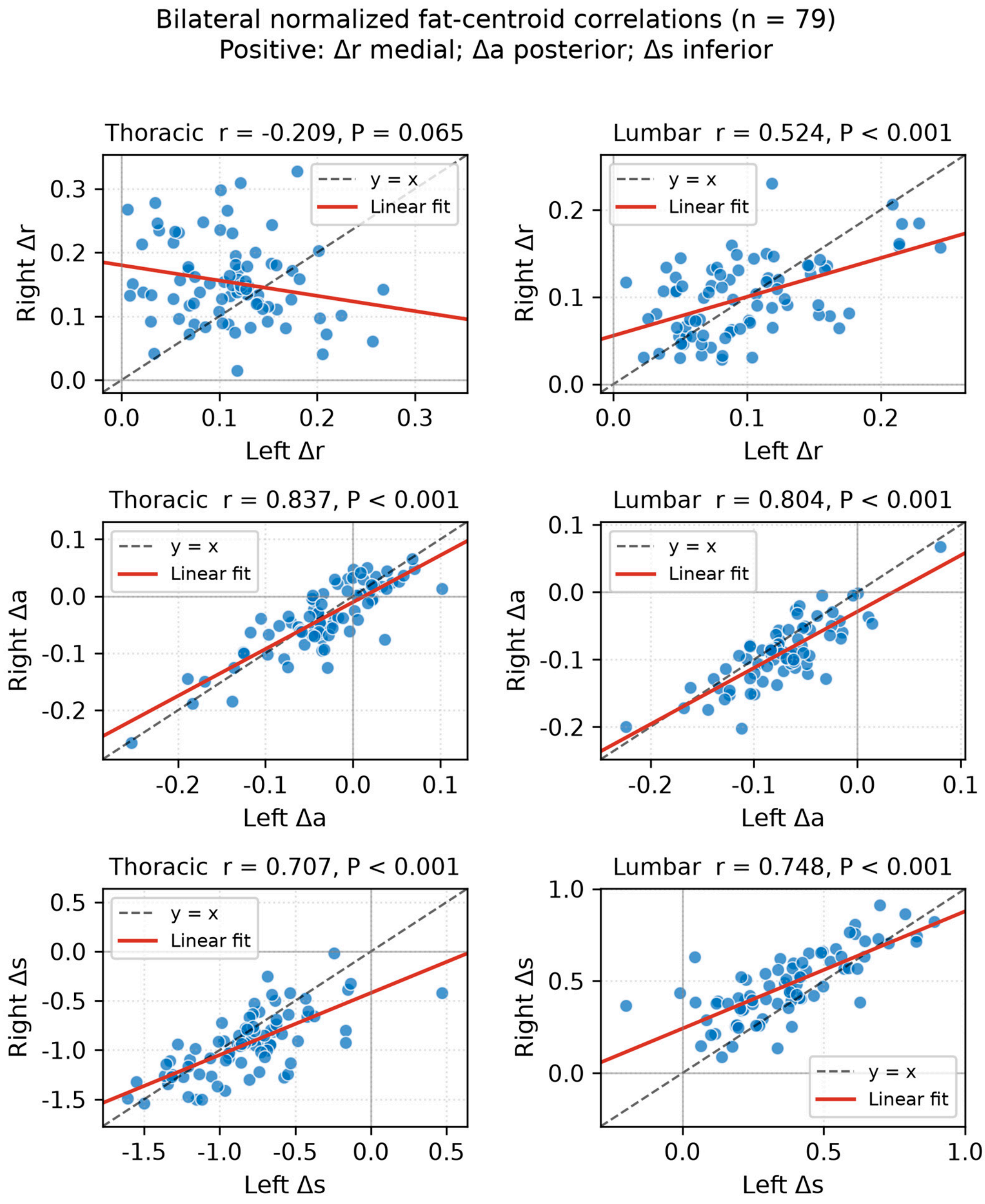

Figure S2. Bilateral relationships of normalized offsets. Rows show Δr, Δa, and Δs; columns show thoracic and lumbar regions (n = 79). Dashed lines indicate equality and red lines indicate linear fits. Pearson r and P are annotated. Correlation does not establish bilateral agreement or reader reliability. These are between-side correlations, not intra-/inter-rater reproducibility or an agreement assessment. Positive directions are medial for Δr, posterior for Δa, and inferior for Δs.

Figure S3

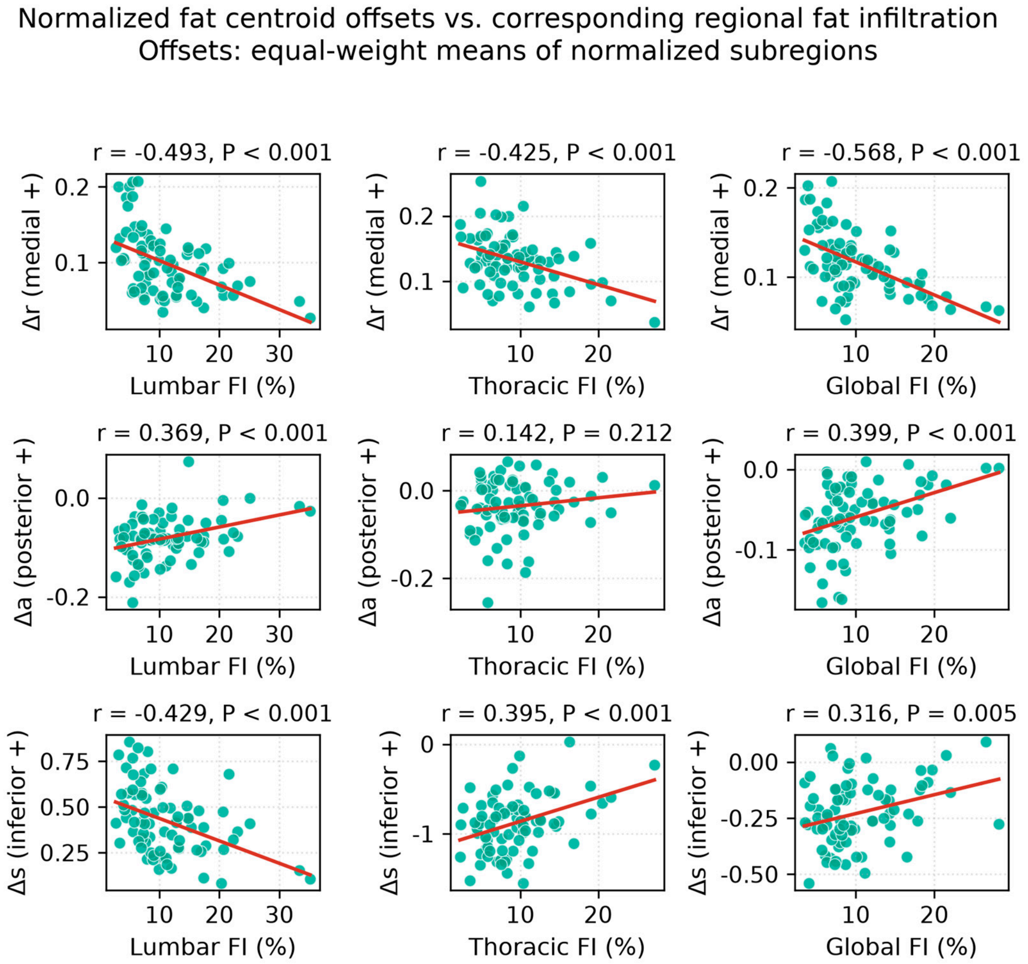

Figure S3. Relationships of normalized offsets with corresponding FI. Rows show Δr, Δa, and Δs; columns show lumbar, thoracic, and global regions (n = 79). Red lines are linear fits. Pearson r and P are annotated. Positive offsets indicate medial, posterior, and inferior positions relative to the fat-inclusive whole compartment.

Figure S4

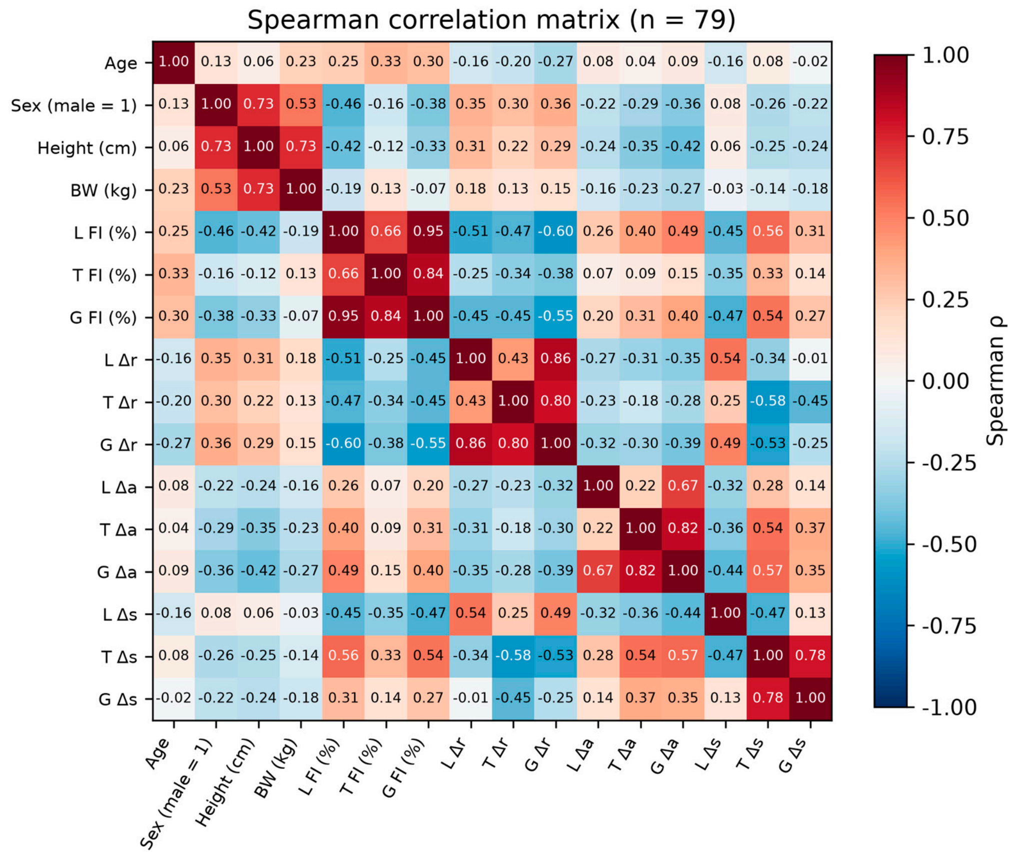

Figure S4. Spearman correlation matrix of explanatory variables. Cells show pairwise Spearman ρ among demographic variables, FI, and normalized offsets (n = 79). Red and blue indicate positive and negative correlations; intensity reflects magnitude, not significance. The matrix summarizes correlation coefficients. Strength outcomes are not included. L, T, and G denote lumbar, thoracic, and global; BW is body weight. Height is in cm, BW in kg, and FI in %. Offsets are dimensionless, with Δr medial-positive, Δa posterior-positive, and Δs inferior-positive.

Table S1. Descriptive statistics.

**Variable**	**n**	**Mean ± SD**	**Median [Q1, Q3]**	**Range**
Age (years)	79	69.620 ± 4.121	69.000 [68.000, 73.000]	60.000–79.000
Sex (male = 1)	79	0.532 ± 0.502	1.000 [0.000, 1.000]	0.000–1.000
Height (cm)	79	163.316 ± 7.793	163.000 [157.500, 170.000]	149.000–182.000
Body weight (kg)	79	66.418 ± 11.250	66.000 [58.500, 74.500]	46.000–91.000
BMI (kg/m²)	79	24.774 ± 2.951	24.558 [22.698, 26.306]	19.228–33.203
Relative extension peak torque (Nm/kg)	79	1.003 ± 0.462	0.900 [0.700, 1.300]	0.200–2.400
Relative flexion peak torque (Nm/kg)	79	0.751 ± 0.345	0.700 [0.500, 0.900]	0.200–1.800
Absolute extension peak torque (Nm)	79	65.699 ± 31.582	61.100 [43.050, 83.550]	13.900–158.900
Absolute flexion peak torque (Nm)	79	49.767 ± 25.534	45.100 [32.750, 59.750]	9.600–126.000

Variable	n	Mean ± SD	Median [Q1, Q3]	Range
Lumbar total volume (cm³)	79	511.062 ± 103.032	499.691 [441.166, 560.832]	303.533–806.342
Lumbar fat volume (cm³)	79	53.222 ± 26.598	44.919 [34.715, 65.558]	17.605–124.922
Lumbar non-fat volume (cm³)	79	457.840 ± 110.387	440.445 [391.069, 522.315]	214.971–768.273
Lumbar FI (%)	79	11.031 ± 6.414	9.062 [6.915, 13.256]	2.733–35.090
Lumbar Δr	79	0.099 ± 0.042	0.093 [0.066, 0.121]	0.027–0.208
Lumbar Δa	79	-0.081 ± 0.043	-0.081 [-0.100, -0.062]	-0.212–0.074
Lumbar Δs	79	0.425 ± 0.184	0.412 [0.279, 0.557]	0.084–0.856
Thoracic total volume (cm³)	79	365.311 ± 83.638	362.052 [300.500, 424.994]	222.784–588.364
Thoracic fat volume (cm³)	79	33.283 ± 17.753	31.624 [21.912, 39.399]	6.801–122.840
Thoracic non-fat volume (cm³)	79	332.028 ± 81.788	327.399 [260.166, 397.889]	198.317–543.844
Thoracic FI (%)	79	9.328 ± 4.636	8.474 [6.060, 11.077]	2.250–27.215
Thoracic Δr	79	0.132 ± 0.038	0.133 [0.108, 0.152]	0.037–0.254
Thoracic Δa	79	-0.036 ± 0.060	-0.034 [-0.061, 0.009]	-0.255–0.067
Thoracic Δs	79	-0.879 ± 0.315	-0.863 [-1.092, -0.702]	-1.549–0.025
Global total volume (cm³)	79	876.374 ± 177.503	864.598 [742.161, 981.727]	539.196–1294.921
Global fat volume (cm³)	79	86.505 ± 40.162	72.891 [59.854, 102.655]	36.789–243.319
Global non-fat volume (cm³)	79	789.869 ± 182.859	783.388 [659.106, 906.672]	439.640–1228.810
Global FI (%)	79	10.313 ± 5.259	8.712 [6.854, 13.189]	3.454–28.198
Global Δr	79	0.116 ± 0.034	0.113 [0.093, 0.131]	0.052–0.208
Global Δa	79	-0.058 ± 0.040	-0.060 [-0.083, -0.028]	-0.166–0.010
Global Δs	79	-0.227 ± 0.140	-0.245 [-0.303, -0.124]	-0.539–0.090
Thoracic left total volume (cm³)	79	185.985 ± 41.706	182.722 [153.513, 218.141]	112.930–295.757
Thoracic left fat volume (cm³)	79	16.388 ± 8.855	15.293 [10.051, 20.254]	3.489–61.702
Thoracic left non-fat volume (cm³)	79	169.597 ± 41.182	165.921 [133.812, 200.570]	92.863–272.964
Thoracic left FI (%)	79	9.048 ± 4.673	8.193 [5.854, 10.493]	1.817–27.667
Thoracic left Δr	79	0.111 ± 0.056	0.113 [0.069, 0.139]	0.006–0.267
Thoracic left Δa	79	-0.034 ± 0.063	-0.034 [-0.059, 0.009]	-0.253–0.102
Thoracic left Δs	79	-0.821 ± 0.360	-0.803 [-1.036, -0.639]	-1.608–0.470
Thoracic right total volume (cm³)	79	179.326 ± 42.387	177.514 [145.856, 207.613]	109.854–292.607
Thoracic right fat volume (cm³)	79	16.894 ± 9.182	14.857 [11.147, 20.900]	3.313–61.138
Thoracic right non-fat volume (cm³)	79	162.431 ± 41.096	160.743 [128.726, 193.208]	94.100–270.879
Thoracic right FI (%)	79	9.627 ± 4.829	8.611 [6.364, 11.519]	2.153–26.773
Thoracic right Δr	79	0.153 ± 0.065	0.143 [0.111, 0.182]	0.015–0.328
Thoracic right Δa	79	-0.038 ± 0.062	-0.038 [-0.068, 0.011]	-0.258–0.066

Variable	n	Mean ± SD	Median [Q1, Q3]	Range
Thoracic right Δs	79	-0.937 ± 0.321	-0.936 [-1.154, -0.750]	-1.542–-0.014
Lumbar left total volume (cm³)	79	258.617 ± 52.586	251.834 [223.934, 284.774]	149.855–405.908
Lumbar left fat volume (cm³)	79	24.517 ± 12.412	21.184 [15.377, 30.238]	7.504–58.573
Lumbar left non-fat volume (cm³)	79	234.100 ± 56.103	226.343 [196.528, 271.565]	108.770–388.979
Lumbar left FI (%)	79	10.075 ± 5.874	8.298 [5.790, 13.140]	2.265–32.690
Lumbar left Δr	79	0.099 ± 0.052	0.088 [0.058, 0.123]	0.009–0.244
Lumbar left Δa	79	-0.072 ± 0.044	-0.072 [-0.094, -0.053]	-0.224–0.080
Lumbar left Δs	79	0.371 ± 0.213	0.380 [0.213, 0.496]	-0.200–0.890
Lumbar right total volume (cm³)	79	252.445 ± 51.153	246.229 [215.279, 282.233]	153.678–400.434
Lumbar right fat volume (cm³)	79	28.705 ± 14.759	24.751 [17.983, 34.333]	10.101–76.010
Lumbar right non-fat volume (cm³)	79	223.740 ± 54.951	217.179 [189.484, 256.886]	106.201–379.294
Lumbar right FI (%)	79	12.006 ± 7.124	10.625 [6.885, 15.102]	3.210–39.852
Lumbar right Δr	79	0.100 ± 0.044	0.099 [0.065, 0.132]	0.028–0.230
Lumbar right Δa	79	-0.089 ± 0.046	-0.090 [-0.113, -0.063]	-0.202–0.067
Lumbar right Δs	79	0.478 ± 0.181	0.447 [0.376, 0.604]	0.089–0.911

All summaries are recomputed after excluding age < 60. Centroid offsets are dimensionless except where explicitly stated. Non-fat volume is the complement of thresholded fat within the combined compartment, not independently measured contractile muscle volume. Panel B below supplies the previously missing paired comparisons.

Table S1, panel B. Paired bilateral comparisons (right minus left; n = 79).

Region	Variable	R–L mean [95% CI]	Paired t P	Wilcoxon P	q
Lumbar	FI (%)	1.931 [1.344, 2.519]	<0.001	<0.001	<0.001
Lumbar	Total volume (cm3)	−6.173 [−8.902, −3.443]	<0.001	<0.001	<0.001
Lumbar	Fat volume (cm3)	4.188 [2.838, 5.538]	<0.001	<0.001	<0.001
Lumbar	Δr	0.001 [−0.010, 0.012]	0.862	0.961	0.961
Lumbar	Δa	−0.017 [−0.023, −0.011]	<0.001	<0.001	<0.001
Lumbar	Δs	0.107 [0.075, 0.139]	<0.001	<0.001	<0.001
Thoracic	FI (%)	0.579 [0.126, 1.032]	0.013	<0.001	0.001
Thoracic	Total volume (cm3)	−6.660 [−8.622, −4.697]	<0.001	<0.001	<0.001
Thoracic	Fat volume (cm3)	0.506 [−0.212, 1.225]	0.165	0.147	0.176
Thoracic	Δr	0.043 [0.022, 0.064]	<0.001	<0.001	<0.001
Thoracic	Δa	−0.004 [−0.012, 0.003]	0.264	0.455	0.496
Thoracic	Δs	−0.116 [−0.175, −0.057]	<0.001	<0.001	0.001

Paired t intervals describe the mean difference; FI differences are in percentage points. Wilcoxon signed-rank tests assess paired differences without a normality assumption. Secondary q values apply to the 12 Wilcoxon comparisons, not to the primary regression families.

Table S2. All primary offset coefficients, outcome-specific FDR q values, and whole-model adjusted R^2.

Outcome	Model	Variable	n	β [95% CI]	P	q	Adj. R^2
Extension	M1	Lumbar Δr	79	0.215 [−0.036, 0.466]	0.092	0.277	0.099
Extension	M2	Lumbar Δa	79	−0.048 [−0.285, 0.188]	0.686	0.826	0.066
Extension	M3	Lumbar Δs	79	0.089 [−0.153, 0.330]	0.466	0.826	0.071
Extension	M4	Thoracic Δr	79	0.295 [0.053, 0.536]	0.018	0.079	0.121
Extension	M5	Thoracic Δa	79	−0.027 [−0.258, 0.204]	0.815	0.826	0.051
Extension	M6	Thoracic Δs	79	−0.141 [−0.385, 0.104]	0.255	0.573	0.067
Extension	M7	Global Δr	79	0.322 [0.061, 0.583]	0.016	0.079	0.139
Extension	M8	Global Δa	79	−0.027 [−0.273, 0.218]	0.826	0.826	0.069
Extension	M9	Global Δs	79	−0.040 [−0.272, 0.193]	0.735	0.826	0.070
Flexion	M1	Lumbar Δr	79	0.261 [0.028, 0.495]	0.029	0.130	0.221
Flexion	M2	Lumbar Δa	79	−0.071 [−0.293, 0.152]	0.530	0.794	0.173
Flexion	M3	Lumbar Δs	79	0.144 [−0.082, 0.370]	0.209	0.471	0.187
Flexion	M4	Thoracic Δr	79	0.221 [−0.008, 0.450]	0.058	0.174	0.213
Flexion	M5	Thoracic Δa	79	−0.001 [−0.217, 0.214]	0.990	0.990	0.173
Flexion	M6	Thoracic Δs	79	−0.125 [−0.353, 0.103]	0.277	0.499	0.186
Flexion	M7	Global Δr	79	0.310 [0.065, 0.555]	0.014	0.124	0.244
Flexion	M8	Global Δa	79	−0.020 [−0.250, 0.211]	0.866	0.990	0.179
Flexion	M9	Global Δs	79	−0.012 [−0.231, 0.206]	0.912	0.990	0.178

Table S2, panel B. Corresponding nonstandardized coefficients.

Outcome	Model	n	B [95% CI]	SE
Extension	M1	79	2.352 [−0.397, 5.101]	1.380
Extension	M2	79	−0.524 [−3.092, 2.044]	1.289
Extension	M3	79	0.223 [−0.383, 0.829]	0.304
Extension	M4	79	3.557 [0.637, 6.476]	1.465
Extension	M5	79	−0.211 [−2.003, 1.581]	0.899
Extension	M6	79	−0.206 [−0.565, 0.152]	0.180
Extension	M7	79	4.333 [0.822, 7.845]	1.762
Extension	M8	79	−0.314 [−3.144, 2.516]	1.420
Extension	M9	79	−0.130 [−0.895, 0.635]	0.384
Flexion	M1	79	2.134 [0.225, 4.043]	0.958
Flexion	M2	79	−0.572 [−2.376, 1.232]	0.906
Flexion	M3	79	0.269 [−0.154, 0.693]	0.213
Flexion	M4	79	1.995 [−0.068, 4.058]	1.035
Flexion	M5	79	−0.008 [−1.257, 1.242]	0.627
Flexion	M6	79	−0.137 [−0.387, 0.113]	0.125
Flexion	M7	79	3.114 [0.656, 5.571]	1.233
Flexion	M8	79	−0.168 [−2.153, 1.816]	0.996
Flexion	M9	79	−0.030 [−0.567, 0.507]	0.269

Each model includes age, sex, corresponding FI (%), and one normalized offset. n = 79. Conventional t-based coefficient-wise 95% CIs are not simultaneous intervals. BH families contain all nine offsets

for flexion and all nine for extension, separately. B is in Nm/kg per dimensionless offset unit; β is standardized. Adjusted R^2 describes the complete model, not the offset alone.

Table S3. Representative FI terms: standardized multivariable linear regression coefficients.

Outcome	Model	Variable	n	β [95% CI]	P
Extension	M1	Lumbar FI (%)	79	−0.163 [−0.444, 0.117]	0.250
Extension	M4	Thoracic FI (%)	79	−0.085 [−0.333, 0.163]	0.498
Extension	M7	Global FI (%)	79	−0.103 [−0.381, 0.176]	0.466
Flexion	M1	Lumbar FI (%)	79	−0.101 [−0.362, 0.160]	0.444
Flexion	M4	Thoracic FI (%)	79	−0.121 [−0.355, 0.114]	0.308
Flexion	M7	Global FI (%)	79	−0.083 [−0.345, 0.178]	0.527

Table S3, panel B. Corresponding nonstandardized coefficients.

Outcome	Model	n	B [95% CI]	SE
Extension	M1	79	−0.012 [−0.032, 0.008]	0.010
Extension	M4	79	−0.008 [−0.033, 0.016]	0.012
Extension	M7	79	−0.009 [−0.033, 0.015]	0.012
Flexion	M1	79	−0.005 [−0.019, 0.009]	0.007
Flexion	M4	79	−0.009 [−0.026, 0.008]	0.009
Flexion	M7	79	−0.005 [−0.023, 0.012]	0.009

Representative FI covariate terms are from M1/M4/M7, adjusted for age, sex, and corresponding Δr (n = 79). FI estimates were not added to the nine-offset FDR families. B is Nm/kg per percentage-point FI; β and its intervals are standardized.

Table S4. Simultaneous left/right multivariable linear regression models: all axes and both regions.

Outcome	Variable	n	β [95% CI]	P
Extension	Lumbar Δr Left	79	0.399 [0.153, 0.645]	0.002
Extension	Lumbar Δr Right	79	−0.204 [−0.469, 0.061]	0.129
Extension	Lumbar Δa Left	79	−0.078 [−0.457, 0.301]	0.683
Extension	Lumbar Δa Right	79	0.026 [−0.347, 0.399]	0.890
Extension	Lumbar Δs Left	79	0.323 [−0.016, 0.662]	0.061
Extension	Lumbar Δs Right	79	−0.240 [−0.584, 0.104]	0.168
Extension	Thoracic Δr Left	79	0.242 [0.007, 0.477]	0.044
Extension	Thoracic Δr Right	79	0.230 [−0.010, 0.469]	0.060
Extension	Thoracic Δa Left	79	0.038 [−0.367, 0.442]	0.853
Extension	Thoracic Δa Right	79	−0.067 [−0.478, 0.344]	0.746
Extension	Thoracic Δs Left	79	−0.097 [−0.411, 0.217]	0.539
Extension	Thoracic Δs Right	79	−0.054 [−0.379, 0.271]	0.742
Flexion	Lumbar Δr Left	79	0.360 [0.127, 0.592]	0.003
Flexion	Lumbar Δr Right	79	−0.099 [−0.350, 0.152]	0.434
Flexion	Lumbar Δa Left	79	−0.091 [−0.447, 0.266]	0.614
Flexion	Lumbar Δa Right	79	0.015 [−0.336, 0.366]	0.932
Flexion	Lumbar Δs Left	79	0.251 [−0.070, 0.572]	0.123
Flexion	Lumbar Δs Right	79	−0.104 [−0.430, 0.221]	0.524

Outcome	Variable	n	β [95% CI]	P
Flexion	Thoracic Δr Left	79	0.164 [−0.058, 0.387]	0.146
Flexion	Thoracic Δr Right	79	0.186 [−0.041, 0.414]	0.106
Flexion	Thoracic Δa Left	79	−0.006 [−0.384, 0.372]	0.975
Flexion	Thoracic Δa Right	79	0.005 [−0.379, 0.389]	0.981
Flexion	Thoracic Δs Left	79	−0.043 [−0.336, 0.251]	0.774
Flexion	Thoracic Δs Right	79	−0.095 [−0.399, 0.209]	0.535

Left and right offsets are entered jointly with age, sex, and corresponding FI (n = 79). These are conditional coefficients, not independent participant groups. Direct equal-slope contrasts are supplied in Table S11; significance on only one side is not evidence of a difference.

Figure S5

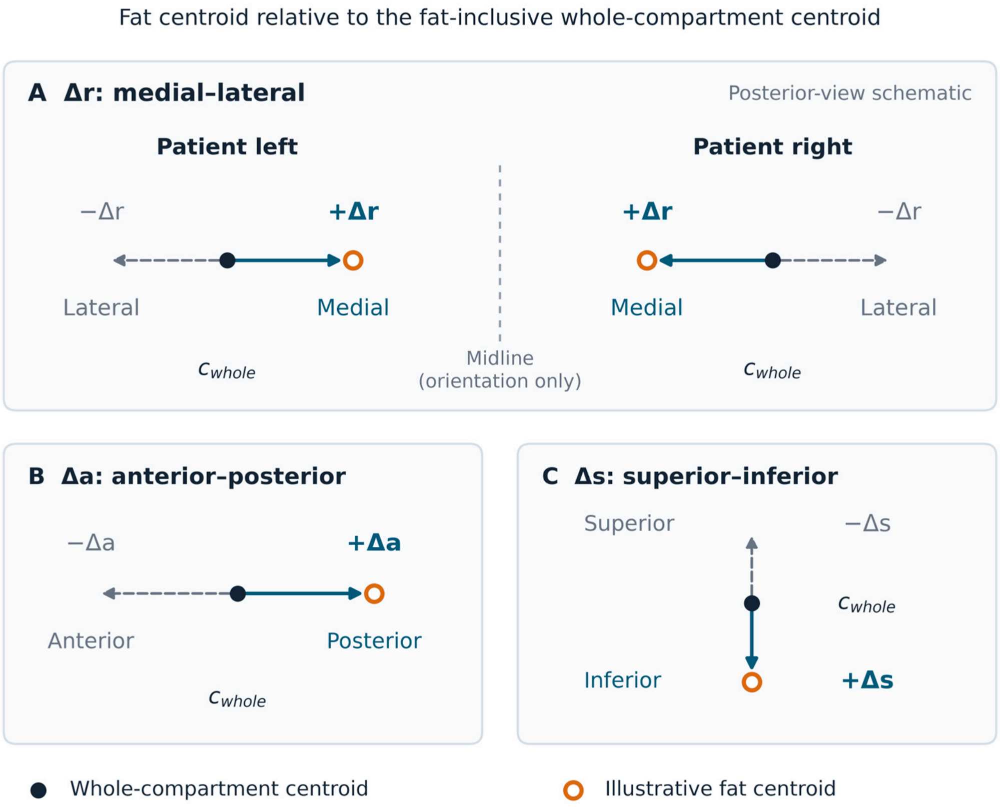

Figure S5. Anatomical direction key for normalized fat-centroid offsets. Black dots denote the fat-inclusive whole-compartment centroid; orange circles are hypothetical positive-direction fat-centroid examples, not participant data. Arrows represent positional differences, not tissue movement. (A) Positive Δr is medial and negative Δr lateral on both sides. The dashed midline is an orientation cue only, not the measurement reference. (B) Positive Δa is posterior and negative Δa anterior. (C) Positive Δs is inferior and negative Δs superior. Zero means no difference along the corresponding axis. Reported offsets are dimensionless. Raw RAS components are right-, anterior-, and superior-positive before correction; r/a/s axis labels are retained afterward. Schematics are not anatomically scaled.

Table S5. Primary regression diagnostics.

**Outcome**	**Model**	**BP P**	**Residual SW P**	**Max VIF**	**Max Cook D**	**Cook >4/n**
Extension	M1	0.048	0.803	1.72	0.112	6
Extension	M2	0.174	0.434	1.62	0.117	5
Extension	M3	0.030	0.557	1.78	0.137	7
Extension	M4	0.084	0.994	1.37	0.117	7
Extension	M5	0.092	0.283	1.23	0.084	6
Extension	M6	0.026	0.499	1.41	0.127	3
Extension	M7	0.104	0.983	1.77	0.120	6
Extension	M8	0.129	0.372	1.56	0.091	5
Extension	M9	0.112	0.443	1.56	0.068	5
Flexion	M1	<0.001	0.237	1.72	0.244	8
Flexion	M2	0.042	0.184	1.62	0.238	5
Flexion	M3	<0.001	0.496	1.78	0.274	8
Flexion	M4	0.011	0.568	1.37	0.291	3
Flexion	M5	0.145	0.058	1.23	0.102	5
Flexion	M6	0.025	0.267	1.41	0.266	5
Flexion	M7	0.003	0.643	1.77	0.306	6
Flexion	M8	0.094	0.098	1.56	0.166	7
Flexion	M9	0.121	0.088	1.56	0.122	3

n = 79 in each model. BP is studentized Breusch–Pagan; SW is Shapiro–Wilk on residuals; VIF excludes the intercept. Cook >4/n is a diagnostic flag, not a rule excluding records from primary analyses. Diagnostic P values are not used as proof that assumptions hold or as an estimate-selection rule.

Table S6, panel A. HC3 t-based uncertainty.

**Outcome**	**Model**	**Variable**	**n**	**β [95% CI]**	**P**	**q**
Extension	M1	Lumbar Δr	79	0.215 [−0.128, 0.558]	0.216	0.618
Extension	M2	Lumbar Δa	79	−0.048 [−0.308, 0.211]	0.712	0.826
Extension	M3	Lumbar Δs	79	0.089 [−0.212, 0.390]	0.558	0.826
Extension	M4	Thoracic Δr	79	0.295 [0.036, 0.553]	0.026	0.236
Extension	M5	Thoracic Δa	79	−0.027 [−0.230, 0.176]	0.790	0.826
Extension	M6	Thoracic Δs	79	−0.141 [−0.395, 0.114]	0.275	0.618
Extension	M7	Global Δr	79	0.322 [−0.010, 0.654]	0.057	0.256
Extension	M8	Global Δa	79	−0.027 [−0.273, 0.219]	0.826	0.826
Extension	M9	Global Δs	79	−0.040 [−0.244, 0.165]	0.701	0.826
Flexion	M1	Lumbar Δr	79	0.261 [−0.107, 0.630]	0.162	0.486
Flexion	M2	Lumbar Δa	79	−0.071 [−0.334, 0.193]	0.595	0.892
Flexion	M3	Lumbar Δs	79	0.144 [−0.171, 0.459]	0.366	0.660
Flexion	M4	Thoracic Δr	79	0.221 [−0.069, 0.511]	0.133	0.486
Flexion	M5	Thoracic Δa	79	−0.001 [−0.175, 0.172]	0.988	0.988
Flexion	M6	Thoracic Δs	79	−0.125 [−0.400, 0.150]	0.367	0.660
Flexion	M7	Global Δr	79	0.310 [−0.056, 0.676]	0.096	0.486
Flexion	M8	Global Δa	79	−0.020 [−0.257, 0.218]	0.870	0.988
Flexion	M9	Global Δs	79	−0.012 [−0.191, 0.167]	0.893	0.988

HC3 changes covariance, not coefficients; Student t residual df are retained. BH still covers the nine offset tests for each outcome within this sensitivity variant.

Table S6, panel B. Model-specific influence-omission check.

Outcome	Model	Variable	n	β [95% CI]	P	q
Extension	M1	Lumbar Δr	73	0.200 [−0.051, 0.451]	0.116	0.348
Extension	M2	Lumbar Δa	74	0.069 [−0.178, 0.316]	0.581	0.759
Extension	M3	Lumbar Δs	72	−0.053 [−0.306, 0.199]	0.675	0.759
Extension	M4	Thoracic Δr	72	0.291 [0.070, 0.511]	0.011	0.090
Extension	M5	Thoracic Δa	73	−0.086 [−0.321, 0.148]	0.466	0.759
Extension	M6	Thoracic Δs	76	−0.024 [−0.270, 0.222]	0.847	0.847
Extension	M7	Global Δr	73	0.313 [0.051, 0.575]	0.020	0.090
Extension	M8	Global Δa	74	−0.063 [−0.319, 0.192]	0.622	0.759
Extension	M9	Global Δs	74	0.070 [−0.171, 0.311]	0.565	0.759
Flexion	M1	Lumbar Δr	71	0.351 [0.135, 0.566]	0.002	0.016
Flexion	M2	Lumbar Δa	74	0.049 [−0.164, 0.263]	0.648	0.838
Flexion	M3	Lumbar Δs	71	−0.117 [−0.331, 0.098]	0.282	0.773
Flexion	M4	Thoracic Δr	76	0.106 [−0.116, 0.328]	0.343	0.773
Flexion	M5	Thoracic Δa	74	−0.054 [−0.256, 0.147]	0.593	0.838
Flexion	M6	Thoracic Δs	74	−0.041 [−0.266, 0.184]	0.715	0.838
Flexion	M7	Global Δr	73	0.281 [0.051, 0.511]	0.017	0.078
Flexion	M8	Global Δa	72	0.025 [−0.196, 0.247]	0.820	0.838
Flexion	M9	Global Δs	76	−0.022 [−0.238, 0.194]	0.838	0.838

Each model omits observations with its own Cook D >4/79; resulting samples differ. All 79 participants remain in the primary analysis. These checks do not justify selecting a favorable subset. BH still covers the nine offset tests for each outcome within this sensitivity variant.

Table S7, panel A. Additional BMI and log total-volume adjustment.

Outcome	Model	Variable	n	β [95% CI]	P	q
Extension	M1	Lumbar Δr	79	0.198 [−0.048, 0.444]	0.113	0.340
Extension	M2	Lumbar Δa	79	−0.058 [−0.285, 0.169]	0.613	0.883
Extension	M3	Lumbar Δs	79	0.062 [−0.172, 0.296]	0.598	0.883
Extension	M4	Thoracic Δr	79	0.294 [0.063, 0.525]	0.013	0.098
Extension	M5	Thoracic Δa	79	0.003 [−0.222, 0.228]	0.981	0.981
Extension	M6	Thoracic Δs	79	−0.130 [−0.369, 0.109]	0.281	0.632
Extension	M7	Global Δr	79	0.299 [0.045, 0.552]	0.022	0.098
Extension	M8	Global Δa	79	−0.029 [−0.266, 0.208]	0.807	0.908
Extension	M9	Global Δs	79	−0.046 [−0.271, 0.180]	0.687	0.883
Flexion	M1	Lumbar Δr	79	0.238 [0.002, 0.474]	0.048	0.145
Flexion	M2	Lumbar Δa	79	−0.074 [−0.294, 0.146]	0.503	0.754
Flexion	M3	Lumbar Δs	79	0.122 [−0.103, 0.347]	0.284	0.638
Flexion	M4	Thoracic Δr	79	0.224 [0.002, 0.446]	0.048	0.145
Flexion	M5	Thoracic Δa	79	0.038 [−0.174, 0.251]	0.720	0.926
Flexion	M6	Thoracic Δs	79	−0.094 [−0.321, 0.132]	0.410	0.738
Flexion	M7	Global Δr	79	0.285 [0.043, 0.527]	0.022	0.145
Flexion	M8	Global Δa	79	−0.016 [−0.242, 0.211]	0.890	0.979
Flexion	M9	Global Δs	79	−0.003 [−0.218, 0.213]	0.979	0.979

All models also include age, sex, and corresponding FI. BMI/volume models add BMI and log fat-inclusive regional/global total compartment volume. Absolute-torque models use recorded Nm, not reconstructed torque, and add height and weight. Exact-ratio models retain the original main predictors but recompute Nm/kg from the recorded torque and weight. n = 79; nine-test BH correction per outcome/variant.

Table S7, panel B. Absolute torque with height and weight adjustment.

**Outcome**	**Model**	**Variable**	**n**	**β [95% CI]**	**P**	**q**
Extension	M1	Lumbar Δr	79	0.164 [−0.080, 0.409]	0.185	0.554
Extension	M2	Lumbar Δa	79	−0.082 [−0.310, 0.145]	0.473	0.852
Extension	M3	Lumbar Δs	79	0.061 [−0.173, 0.295]	0.605	0.899
Extension	M4	Thoracic Δr	79	0.275 [0.041, 0.510]	0.022	0.156
Extension	M5	Thoracic Δa	79	−0.005 [−0.234, 0.223]	0.962	0.962
Extension	M6	Thoracic Δs	79	−0.126 [−0.365, 0.113]	0.298	0.670
Extension	M7	Global Δr	79	0.275 [0.020, 0.530]	0.035	0.156
Extension	M8	Global Δa	79	−0.038 [−0.279, 0.204]	0.756	0.899
Extension	M9	Global Δs	79	−0.029 [−0.255, 0.197]	0.799	0.899
Flexion	M1	Lumbar Δr	79	0.190 [−0.036, 0.416]	0.098	0.293
Flexion	M2	Lumbar Δa	79	−0.070 [−0.282, 0.142]	0.515	0.772
Flexion	M3	Lumbar Δs	79	0.110 [−0.107, 0.326]	0.316	0.588
Flexion	M4	Thoracic Δr	79	0.196 [−0.023, 0.415]	0.079	0.293
Flexion	M5	Thoracic Δa	79	0.022 [−0.187, 0.232]	0.832	0.934
Flexion	M6	Thoracic Δs	79	−0.109 [−0.329, 0.111]	0.327	0.588
Flexion	M7	Global Δr	79	0.248 [0.011, 0.484]	0.040	0.293
Flexion	M8	Global Δa	79	−0.009 [−0.233, 0.215]	0.934	0.934
Flexion	M9	Global Δs	79	−0.019 [−0.227, 0.190]	0.860	0.934

All models also include age, sex, and corresponding FI. BMI/volume models add BMI and log fat-inclusive regional/global total compartment volume. Absolute-torque models use recorded Nm, not reconstructed torque, and add height and weight. Exact-ratio models retain the original main predictors but recompute Nm/kg from the recorded torque and weight. n = 79; nine-test BH correction per outcome/variant.

Table S7, panel C. Unrounded absolute-torque/body-weight ratios.

Outcome	Model	Variable	n	β [95% CI]	P	q
Extension	M1	Lumbar Δr	79	0.216 [−0.035, 0.467]	0.090	0.271
Extension	M2	Lumbar Δa	79	−0.056 [−0.293, 0.180]	0.638	0.782
Extension	M3	Lumbar Δs	79	0.104 [−0.137, 0.345]	0.393	0.707
Extension	M4	Thoracic Δr	79	0.311 [0.071, 0.551]	0.012	0.056
Extension	M5	Thoracic Δa	79	−0.032 [−0.263, 0.198]	0.782	0.782
Extension	M6	Thoracic Δs	79	−0.148 [−0.392, 0.096]	0.230	0.517
Extension	M7	Global Δr	79	0.334 [0.074, 0.594]	0.012	0.056
Extension	M8	Global Δa	79	−0.037 [−0.282, 0.209]	0.768	0.782
Extension	M9	Global Δs	79	−0.040 [−0.273, 0.192]	0.729	0.782
Flexion	M1	Lumbar Δr	79	0.257 [0.023, 0.491]	0.032	0.142
Flexion	M2	Lumbar Δa	79	−0.069 [−0.292, 0.154]	0.538	0.808
Flexion	M3	Lumbar Δs	79	0.155 [−0.071, 0.380]	0.177	0.397
Flexion	M4	Thoracic Δr	79	0.221 [−0.008, 0.451]	0.059	0.176
Flexion	M5	Thoracic Δa	79	−0.008 [−0.225, 0.208]	0.940	0.940
Flexion	M6	Thoracic Δs	79	−0.136 [−0.364, 0.093]	0.240	0.432
Flexion	M7	Global Δr	79	0.307 [0.061, 0.552]	0.015	0.135
Flexion	M8	Global Δa	79	−0.025 [−0.256, 0.205]	0.827	0.940
Flexion	M9	Global Δs	79	−0.012 [−0.231, 0.207]	0.914	0.940

All models also include age, sex, and corresponding FI. BMI/volume models add BMI and log fat-inclusive regional/global total compartment volume. Absolute-torque models use recorded Nm, not reconstructed torque, and add height and weight. Exact-ratio models retain the original main predictors but recompute Nm/kg from the recorded torque and weight. n = 79; nine-test BH correction per outcome/variant.

Table S8, panel A. Direction-corrected offsets in millimeters.

**Outcome**	**Model**	**Variable**	**n**	**β [95% CI]**	**P**	**q**
Extension	M1	Lumbar Δr	79	0.201 [−0.060, 0.462]	0.130	0.389
Extension	M2	Lumbar Δa	79	−0.048 [−0.292, 0.197]	0.699	0.882
Extension	M3	Lumbar Δs	79	0.082 [−0.166, 0.330]	0.513	0.882
Extension	M4	Thoracic Δr	79	0.287 [0.030, 0.545]	0.029	0.161
Extension	M5	Thoracic Δa	79	−0.022 [−0.256, 0.212]	0.854	0.882
Extension	M6	Thoracic Δs	79	−0.128 [−0.384, 0.128]	0.322	0.725
Extension	M7	Global Δr	79	0.300 [0.021, 0.580]	0.036	0.161
Extension	M8	Global Δa	79	−0.026 [−0.281, 0.228]	0.836	0.882
Extension	M9	Global Δs	79	−0.018 [−0.252, 0.217]	0.882	0.882
Flexion	M1	Lumbar Δr	79	0.266 [0.023, 0.508]	0.032	0.100
Flexion	M2	Lumbar Δa	79	−0.084 [−0.313, 0.146]	0.470	0.705
Flexion	M3	Lumbar Δs	79	0.160 [−0.072, 0.391]	0.174	0.379
Flexion	M4	Thoracic Δr	79	0.262 [0.021, 0.502]	0.033	0.100
Flexion	M5	Thoracic Δa	79	−0.003 [−0.221, 0.215]	0.980	0.980
Flexion	M6	Thoracic Δs	79	−0.151 [−0.389, 0.087]	0.211	0.379
Flexion	M7	Global Δr	79	0.338 [0.079, 0.597]	0.011	0.100
Flexion	M8	Global Δa	79	−0.034 [−0.273, 0.205]	0.776	0.980
Flexion	M9	Global Δs	79	−0.013 [−0.233, 0.208]	0.909	0.980

Age, sex, and corresponding FI are retained. Raw-mm offsets are corrected before equal-weight averaging. Alternative lengths are 10*cuberoot(Vtot−Vfat) mm, used with the original whole-reference difference; this is not axis-specific geometric normalization. Volume weighting uses Vtot times each already-normalized side value divided by summed Vtot, not a pooled centroid. n = 79; nine-test BH correction per outcome/variant.

Table S8, panel B. Non-fat-volume cube-root length.

**Outcome**	**Model**	**Variable**	**n**	**β [95% CI]**	**P**	**q**
Extension	M1	Lumbar Δr	79	0.208 [−0.038, 0.455]	0.097	0.290
Extension	M2	Lumbar Δa	79	−0.046 [−0.280, 0.188]	0.696	0.826
Extension	M3	Lumbar Δs	79	0.084 [−0.153, 0.322]	0.480	0.826
Extension	M4	Thoracic Δr	79	0.285 [0.048, 0.522]	0.019	0.086
Extension	M5	Thoracic Δa	79	−0.028 [−0.259, 0.203]	0.808	0.826
Extension	M6	Thoracic Δs	79	−0.136 [−0.377, 0.104]	0.263	0.591
Extension	M7	Global Δr	79	0.312 [0.057, 0.566]	0.017	0.086
Extension	M8	Global Δa	79	−0.027 [−0.270, 0.216]	0.826	0.826
Extension	M9	Global Δs	79	−0.039 [−0.271, 0.193]	0.739	0.826
Flexion	M1	Lumbar Δr	79	0.253 [0.024, 0.483]	0.031	0.139
Flexion	M2	Lumbar Δa	79	−0.068 [−0.288, 0.152]	0.539	0.809
Flexion	M3	Lumbar Δs	79	0.139 [−0.083, 0.361]	0.217	0.489
Flexion	M4	Thoracic Δr	79	0.216 [−0.008, 0.440]	0.059	0.177
Flexion	M5	Thoracic Δa	79	−0.002 [−0.218, 0.213]	0.983	0.983
Flexion	M6	Thoracic Δs	79	−0.122 [−0.347, 0.103]	0.283	0.510
Flexion	M7	Global Δr	79	0.301 [0.062, 0.539]	0.014	0.128
Flexion	M8	Global Δa	79	−0.020 [−0.248, 0.209]	0.865	0.983
Flexion	M9	Global Δs	79	−0.011 [−0.229, 0.207]	0.923	0.983

Age, sex, and corresponding FI are retained. Raw-mm offsets are corrected before equal-weight averaging. Alternative lengths are 10*cuberoot(Vtot−Vfat) mm, used with the original whole-reference difference; this is not axis-specific geometric normalization. Volume weighting uses Vtot times each already-normalized side value divided by summed Vtot, not a pooled centroid. n = 79; nine-test BH correction per outcome/variant.

Table S8, panel C. Total-volume-weighted normalized offsets.

**Outcome**	**Model**	**Variable**	**n**	**β [95% CI]**	**P**	**q**
Extension	M1	Lumbar Δr	79	0.217 [−0.034, 0.468]	0.089	0.266
Extension	M2	Lumbar Δa	79	−0.049 [−0.286, 0.187]	0.678	0.907
Extension	M3	Lumbar Δs	79	0.090 [−0.151, 0.331]	0.460	0.828
Extension	M4	Thoracic Δr	79	0.293 [0.051, 0.535]	0.018	0.092
Extension	M5	Thoracic Δa	79	−0.027 [−0.258, 0.204]	0.815	0.907
Extension	M6	Thoracic Δs	79	−0.140 [−0.384, 0.104]	0.256	0.575
Extension	M7	Global Δr	79	0.310 [0.049, 0.570]	0.021	0.092
Extension	M8	Global Δa	79	−0.037 [−0.284, 0.209]	0.764	0.907
Extension	M9	Global Δs	79	−0.013 [−0.238, 0.212]	0.907	0.907
Flexion	M1	Lumbar Δr	79	0.261 [0.028, 0.495]	0.029	0.130
Flexion	M2	Lumbar Δa	79	−0.073 [−0.295, 0.150]	0.518	0.776
Flexion	M3	Lumbar Δs	79	0.144 [−0.082, 0.370]	0.208	0.469
Flexion	M4	Thoracic Δr	79	0.219 [−0.009, 0.448]	0.060	0.180
Flexion	M5	Thoracic Δa	79	−0.001 [−0.217, 0.215]	0.992	0.992
Flexion	M6	Thoracic Δs	79	−0.124 [−0.352, 0.104]	0.283	0.509
Flexion	M7	Global Δr	79	0.312 [0.069, 0.555]	0.013	0.113
Flexion	M8	Global Δa	79	−0.029 [−0.260, 0.203]	0.805	0.992
Flexion	M9	Global Δs	79	0.008 [−0.204, 0.219]	0.941	0.992

Age, sex, and corresponding FI are retained. Raw-mm offsets are corrected before equal-weight averaging. Alternative lengths are 10*cuberoot(Vtot−Vfat) mm, used with the original whole-reference difference; this is not axis-specific geometric normalization. Volume weighting uses Vtot times each already-normalized side value divided by summed Vtot, not a pooled centroid. n = 79; nine-test BH correction per outcome/variant.

Table S9, panel A. Reference-centroid sensitivity of FI–offset correlations.

Region	Axis	Whole r	Whole P	Complement r	Complement P
Lumbar	Δr	−0.493	<0.001	−0.384	<0.001
Lumbar	Δa	0.369	<0.001	0.269	0.017
Lumbar	Δs	−0.429	<0.001	−0.300	0.007
Thoracic	Δr	−0.425	<0.001	−0.296	0.008
Thoracic	Δa	0.142	0.212	0.125	0.271
Thoracic	Δs	0.395	<0.001	0.292	0.009
Global	Δr	−0.568	<0.001	−0.456	<0.001
Global	Δa	0.399	<0.001	0.345	0.002
Global	Δs	0.316	0.005	0.295	0.008

n = 79. These Pearson comparisons are descriptive. cnonfat = (cwhole−f*cfat)/(1−f), with f=Vfat/Vtot in each nested, same-grid mask. The corrected fat-to-whole difference equals (1−f) times the fat-to-complement difference. Original total-volume lengths and equal weights are retained. The complement contains all non-fat voxels in the mask, not a separately validated contractile muscle segmentation.

Table S9, panel B. Reconstructed non-fat-reference offset regressions.

**Outcome**	**Model**	**Variable**	**n**	**β [95% CI]**	**P**	**q**
Extension	M1	Lumbar Δr	79	0.196 [−0.041, 0.433]	0.104	0.313
Extension	M2	Lumbar Δa	79	−0.042 [−0.270, 0.186]	0.713	0.824
Extension	M3	Lumbar Δs	79	0.077 [−0.152, 0.306]	0.505	0.824
Extension	M4	Thoracic Δr	79	0.269 [0.039, 0.498]	0.022	0.101
Extension	M5	Thoracic Δa	79	−0.030 [−0.261, 0.200]	0.795	0.824
Extension	M6	Thoracic Δs	79	−0.129 [−0.363, 0.106]	0.278	0.624
Extension	M7	Global Δr	79	0.292 [0.050, 0.534]	0.019	0.101
Extension	M8	Global Δa	79	−0.027 [−0.265, 0.212]	0.824	0.824
Extension	M9	Global Δs	79	−0.038 [−0.270, 0.194]	0.745	0.824
Flexion	M1	Lumbar Δr	79	0.238 [0.018, 0.459]	0.035	0.156
Flexion	M2	Lumbar Δa	79	−0.064 [−0.278, 0.151]	0.555	0.832
Flexion	M3	Lumbar Δs	79	0.130 [−0.085, 0.344]	0.232	0.521
Flexion	M4	Thoracic Δr	79	0.207 [−0.010, 0.424]	0.061	0.182
Flexion	M5	Thoracic Δa	79	−0.004 [−0.220, 0.211]	0.967	0.967
Flexion	M6	Thoracic Δs	79	−0.116 [−0.335, 0.103]	0.294	0.529
Flexion	M7	Global Δr	79	0.283 [0.056, 0.510]	0.015	0.135
Flexion	M8	Global Δa	79	−0.020 [−0.245, 0.204]	0.857	0.967
Flexion	M9	Global Δs	79	−0.008 [−0.225, 0.210]	0.945	0.967

All models adjust for age, sex, and corresponding FI. This algebraic reconstruction addresses the reference mixture, but cannot substitute for independent voxel resegmentation or muscle-specific validation. n = 79; BH covers nine offsets per outcome.

Table S10. Incremental in-sample fit from adding each offset.

**Outcome**	**Model**	**Base R^2**	**Full R^2**	**ΔR^2**	**Partial R^2**	**Full adj. R^2**
Extension	M1	0.112	0.146	0.034	0.038	0.099
Extension	M2	0.112	0.114	0.002	0.002	0.066
Extension	M3	0.112	0.119	0.006	0.007	0.071
Extension	M4	0.099	0.166	0.066	0.074	0.121
Extension	M5	0.099	0.100	0.001	0.001	0.051
Extension	M6	0.099	0.115	0.016	0.017	0.067
Extension	M7	0.116	0.183	0.067	0.076	0.139
Extension	M8	0.116	0.117	0.001	0.001	0.069
Extension	M9	0.116	0.118	0.001	0.002	0.070
Flexion	M1	0.212	0.261	0.050	0.063	0.221
Flexion	M2	0.212	0.216	0.004	0.005	0.173
Flexion	M3	0.212	0.228	0.017	0.021	0.187
Flexion	M4	0.216	0.253	0.037	0.048	0.213
Flexion	M5	0.216	0.216	0.000	0.000	0.173
Flexion	M6	0.216	0.228	0.012	0.016	0.186
Flexion	M7	0.220	0.282	0.062	0.079	0.244
Flexion	M8	0.220	0.221	0.000	0.000	0.179
Flexion	M9	0.220	0.221	0.000	0.000	0.178

Base: age + sex + corresponding FI. Full: base + one offset. All nested comparisons use the same 79 participants. $\Delta R^2 = R^2\text{full} - R^2\text{base}$; partial $R^2 = \Delta R^2/(1 - R^2\text{base})$. Added-predictor significance is the same test reported in Table S2, not a new independent hypothesis. These are sample-level descriptive fit improvements, not cross-validated prediction or established clinical usefulness.

Table S11. Direct conditional left-minus-right offset slope contrasts.

**Outcome**	**Region/axis**	**Bdiff [95% CI]**	**P**	**q**	**HC3 P**
Extension	Lumbar Δr	5.654 [1.506, 9.801]	0.008	0.049	0.014
Extension	Lumbar Δa	−1.084 [−8.396, 6.228]	0.768	0.906	0.777
Extension	Lumbar Δs	1.315 [−0.182, 2.813]	0.084	0.253	0.035
Extension	Thoracic Δr	0.341 [−1.777, 2.459]	0.749	0.906	0.740
Extension	Thoracic Δa	0.781 [−5.008, 6.571]	0.789	0.906	0.795
Extension	Thoracic Δs	−0.047 [−0.839, 0.744]	0.906	0.906	0.901
Flexion	Lumbar Δr	3.144 [0.212, 6.076]	0.036	0.216	0.098
Flexion	Lumbar Δa	−0.825 [−5.962, 4.311]	0.750	0.988	0.763
Flexion	Lumbar Δs	0.606 [−0.452, 1.665]	0.257	0.772	0.270
Flexion	Thoracic Δr	0.012 [−1.486, 1.510]	0.988	0.988	0.988
Flexion	Thoracic Δa	−0.059 [−4.097, 3.979]	0.977	0.988	0.976
Flexion	Thoracic Δs	0.061 [−0.491, 0.613]	0.825	0.988	0.821

Contrasts use unstandardized slopes on the same dimensionless offset scale, in joint left/right models adjusted for age, sex, and corresponding FI. BH q values cover six contrasts per outcome. HC3 P is a covariance sensitivity check, not a separate discovery criterion. n = 79.

Table S12. Intra- and inter-rater repeatability of FI and normalized fat-centroid offsets (n = 15).

A. Intra-rater repeatability

Measure	n	ICC(2,1) (95% CI)	SEM	MDC95
Thoracic FI	15	0.9999 (0.9998–1.0000)	0.0409	0.1135
Lumbar FI	15	0.9996 (0.9917–0.9999)	0.0525	0.1454
Global FI	15	0.9998 (0.9963–1.0000)	0.0383	0.1063
Thoracic Δr	15	0.9979 (0.9939–0.9993)	0.0016	0.0044
Thoracic Δa	15	0.9998 (0.9993–0.9999)	0.0008	0.0022
Thoracic Δs	15	0.9992 (0.9953–0.9998)	0.0081	0.0224
Lumbar Δr	15	0.9979 (0.9938–0.9993)	0.0015	0.0041
Lumbar Δa	15	0.9980 (0.9942–0.9993)	0.0024	0.0068
Lumbar Δs	15	0.9966 (0.9832–0.9990)	0.0074	0.0206
Global Δr	15	0.9987 (0.9963–0.9996)	0.0009	0.0024
Global Δa	15	0.9989 (0.9967–0.9996)	0.0013	0.0035
Global Δs	15	0.9992 (0.9975–0.9997)	0.0050	0.0139

B. Inter-rater repeatability

Measure	n	ICC(2,1) (95% CI)	SEM	MDC95
Thoracic FI	15	0.9999 (0.9998–1.0000)	0.0537	0.1488
Lumbar FI	15	0.9979 (0.9889–0.9994)	0.1694	0.4696
Global FI	15	0.9993 (0.9967–0.9998)	0.1000	0.2772
Thoracic Δr	15	0.9965 (0.9882–0.9989)	0.0019	0.0051
Thoracic Δa	15	0.9997 (0.9990–0.9999)	0.0009	0.0026
Thoracic Δs	15	0.9990 (0.9946–0.9997)	0.0095	0.0263
Lumbar Δr	15	0.9566 (0.8914–0.9745)	0.0083	0.0231
Lumbar Δa	15	0.9838 (0.9540–0.9945)	0.0070	0.0193
Lumbar Δs	15	0.9965 (0.9799–0.9990)	0.0073	0.0202
Global Δr	15	0.9722 (0.9405–0.9905)	0.0038	0.0106
Global Δa	15	0.9918 (0.9765–0.9972)	0.0034	0.0093
Global Δs	15	0.9986 (0.9959–0.9995)	0.0065	0.0179

Workflow. Fifteen participants were randomly selected for repeatability assessment. Both raters reviewed and made minor manual corrections to the same automatically generated initial segmentation, rather than performing separate manual segmentations from scratch. The same rater repeated the assessment after approximately one month under blinded conditions.

Definitions and units. ICC(2,1), two-way random-effects, absolute-agreement, single-measurement intraclass correlation coefficient; CI, confidence interval; FI, fat infiltration; SEM, standard error of measurement; MDC95, minimal detectable change at 95% confidence. FI is reported as %; its SEM and MDC95 are in percentage points. Normalized offsets and their SEM and MDC95 are dimensionless. Positive Δr, Δa and Δs indicate medial, posterior and inferior offsets, respectively. Regional and global offsets are equal-weight means of the normalized subregional values.

Interpretation. These estimates characterize repeatability of the shared-initialization, manually corrected workflow, not independent-from-scratch segmentation agreement or segmentation accuracy. Repeat CT acquisition variability was not assessed. MDC95 is not a minimal clinically important difference. Values are shown to four decimal places; an upper CI limit displayed as 1.0000 does not establish perfect agreement.
